# Shape-Programming in Hyperelasticity Through Differential Growth

**DOI:** 10.1007/s00245-024-10117-6

**Published:** 2024-03-23

**Authors:** Rogelio Ortigosa-Martínez, Jesús Martínez-Frutos, Carlos Mora-Corral, Pablo Pedregal, Francisco Periago

**Affiliations:** 1 Department of Applied Mathematics and Statistics, Technical University of Cartagena, Campus Muralla del Mar, 30202 Cartagena, Murcia Spain; 2 Multiphysics Simulation and Optimization Lab, Technical University of Cartagena, Campus Muralla del Mar, 30202 Cartagena, Murcia Spain; 3 Department of Mathematics, University Autónoma of Madrid, 28049 Madrid, Spain; 4 Instituto de Ciencias Matemáticas, CSIC-UAM-UC3M-UCM, 28049 Madrid, Spain; 5 Department of Mathematics, INEI, University of Castilla-La Mancha, Camilo José Cela, 2, 13071 Ciudad Real, Castilla-La Mancha Spain

**Keywords:** Soft robotics, Differential growth, Hyperelasticity, Shape-programming, Optimal control, Numerical simulation methods, 49J45, 65K10, 74B20, 93A30

## Abstract

This paper is concerned with the growth-driven shape-programming problem, which involves determining a growth tensor that can produce a deformation on a hyperelastic body reaching a given target shape. We consider the two cases of globally compatible growth, where the growth tensor is a deformation gradient over the undeformed domain, and the incompatible one, which discards such hypothesis. We formulate the problem within the framework of optimal control theory in hyperelasticity. The Hausdorff distance is used to quantify dissimilarities between shapes; the complexity of the actuation is incorporated in the cost functional as well. Boundary conditions and external loads are allowed in the state law, thus extending previous works where the stress-free hypothesis turns out to be essential. A rigorous mathematical analysis is then carried out to prove the well-posedness of the problem. The numerical approximation is performed using gradient-based optimisation algorithms. Our main goal in this part is to show the possibility to apply inverse techniques for the numerical approximation of this problem, which allows us to address more generic situations than those covered by analytical approaches. Several numerical experiments for beam-like and shell-type geometries illustrate the performance of the proposed numerical scheme.

## Introduction

Soft robotics is a biologically-inspired groundbreaking technology that aims to mimic mechanical deformations, which take place in humans, animals, or plants, through actuated soft materials: dielectric elastomers or magneto-active polymers, for instance. Several actuation mechanisms, such as fluidic, heat, electric, or magnetic, may be used to control these materials [1]. The range of potential applications of this new generation of robots includes, among many others, medical assistance and ocean exploration [2, 3].

In addition to the development of new manufacturing technologies, mathematical modelling, analysis, and numerical simulation are tools of paramount importance to speed up progress in this field. Being composed of soft matter, nonlinear continuum mechanics is the appropriate physical theory to model the kinematics and dynamics of these materials. However, the mathematical control theory of hyperelastic materials is scarce. Indeed, the first mathematically rigorous study for control problems in the hyperelasticity setting appears to be [4]. More recently, several papers have addressed the control of soft materials from the viewpoints of mathematical analysis and numerical simulation [5–9]. See also [10] for a recent survey.

Growth is another biological process susceptible to being mimicked by artificial soft materials. As a matter of fact, [11] reports on a class of soft pneumatic robots whose movements are driven by growth. As for the mathematical modelling of growth, A. Goriley provides, in his seminal book [12], the required ingredients. Doubtless, the topic of mathematical analysis and numerical simulation of growth control is in its infancy, insofar as the mathematical analysis of soft materials actuated by growth is missing in the literature, and only a few works have addressed the numerical simulation counterpart. In this regard, it is worth mentioning [13, 14]. Both papers tackle the so-called *shape-morphing problem*, where the goal is to find the *growth tensor* that can produce the deformation of a given soft continuum to a desired shape. The paper [13] copes with the complexity of the activation as well, and provides explicit solutions in the case of affine shape changes. In a complementary manner, [14] focuses on the case of shells and also finds analytical solutions under the stress-free assumption.

Fostered by [13, 14], this paper sets up the shape-morphing problem within the framework of optimal control theory. Indeed, the control variable is the growth tensor. We consider both cases in which the growth tensor is globally compatible, meaning that it is a deformation gradient over the undeformed domain, and the incompatible one, where it is no longer a gradient. From the viewpoint of mathematical modelling, the former case is expressed as the composition of two mechanical deformations: one of them accounts for growth and the other one incorporates boundary conditions and other possible effects like external loads. The latter case relies on the theory of Morphoelasticity, where a local elastic tensor restores the compatibility that is lost by the growth tensor. The state variable is the deformation of the actuated soft continuum. As usual in hyperelasticity theory, that deformation is a minimiser of a polyconvex energy functional. The cost function uses the Hausdorff distance to account for dissimilarities between the desired shape and the final configuration. It also includes a term to deal with the complexity of the activation. In our work, we do not neeed the stress-free hypothesis of [14].

The outline of this paper is as follows. Section 2 contains the modelling details. Section 3 performs a rigorous mathematical analysis of the shape-programming problem in the globally compatible case, which is more involved analytically than the incompatible one. More precisely, firstly, we prove that, for a given growth tensor, there exist minimisers of the underlying energy functional. Secondly, we establish the existence of solutions for the optimal control problem. We rely on the Direct Method of Calculus of Variations in both cases. Section 4 straightforwardly extends these existence results to the incompatible case. Eventually, Sect. 5 addresses the numerical approximation of the shape-programming problem. Our purpose here is to show how inverse techniques may be used for the numerical resolution of this problem, thus addressing more generic situations than those covered by analytical approaches. We adopt a pragmatic point of view in this part as we are not concerned about the compatible or incompatible nature of the growth tensor; in practise, this amounts to accept the incompatibility and, hence, the study lies in the theory of Morphoelasticity. Likewise, we take the right Cauchy–Green deformation tensor as the main variable since, by a suitable parametrisation, it highly simplifies the numerical approximation of the problem. We also derive explicit formulae for the gradients of the functional involved, and transfer those to cutting-edge optimization algorithms that use gradients, in particular, the interior-point method, to obtain the desired solutions. Several numerical examples for beam-like and shell-type applications, as well as a problem converting a square into a circular geometry, illustrate the performance of the proposed numerical scheme.

## Problem Setting

### Modelling Differential Growth in Nonlinear Elasticity

Let $\Omega _0\subset \mathbb {R}^N$, $N=2,3$, be an open, bounded and connected domain which represents the reference (or undeformed) configuration of an elastic and soft body. If $\Omega _0$ experiences a growth effect (as happens in plants or in human tissues, for instance), then it changes its size or shape.

There are two ways to understand and model this phenomenon. In the first, we postulate that there is an underlying deformation that produces the growth. Let us then denote by $\varvec{\Phi }_{\textrm{g}}:\Omega _0\rightarrow \mathbb {R}^N$ the deformation mapping induced by this phenomenon, and by $\Omega _{\textrm{g}}:= \varvec{\Phi }_{\textrm{g}} \left( \Omega _0\right)$ the deformed body once growth has taken place. It is assumed that $\varvec{\Phi }_{\textrm{g}}$ is a Sobolev map. Although driven by growth, $\varvec{\Phi }_{\textrm{g}}$ is still assumed to be a mechanical deformation, hence it satisfies the properties required to any such deformation; in particular, it preserves the orientation and does not interpenetrate [15]. Let us denote by $\varvec{G}=\varvec{G}(\varvec{X})$ the deformation gradient tensor associated with $\varvec{\Phi }_{\textrm{g}}$, i.e., $\varvec{G}:= \varvec{\nabla } \varvec{\Phi }_{\textrm{g}}$, where $\varvec{\nabla }$ is the material gradient operator with respect to $\varvec{X}\in \Omega _0$. The orientation-preserving condition is modeled with the constraint

2.1 $$\begin{aligned} \det \varvec{G}(\varvec{X}) >0 \quad \text {for almost every } \varvec{X}\in \Omega _0 , \end{aligned}$$

while the non-interpenetration is modelled by imposing that $\varvec{\Phi }_{\textrm{g}}$ is injective almost everywhere (hereafter abbreviated to *a.e.*), so that the restriction of $\varvec{\Phi }_{\textrm{g}}$ to the complement of a set of measure zero is injective [16].

For the second possibility, and according to the general modelling of growth and morphoelasticity in [12], the postulate that there is an underlying deformation $\varvec{\Phi }_{\textrm{g}}$ responsible for growth is discarded, so the tensor $\varvec{G}$ is not assumed to come from any deformation, though still (2.1) is retained.

Since the first alternative is more involved analytically, we will keep our general discussion (this section and Sect. 3) in that context, and defer some comments on the second one (Sect. 4), once the main analysis has been performed. Even so, numerical experiments in Sect. 5 are explored in the morphoelasticity scenario.

Since $\Omega _\textrm{g}$ is an elastic and soft material, it has an internal elastic energy, which is able to induce a new deformation $\varvec{\Phi }_{\textrm{e}}$ on the body $\Omega _\textrm{g}$. For this initial exposition of the problem, we can think that $\varvec{\Phi }_{\textrm{e}}$ is Lipschitz, but this assumption is not necessary in the analysis. Although, in principle, the elastic energy might depend on the configuration $\Omega _\textrm{g}$ and the growth deformation $\varvec{\Phi }_{\textrm{g}}$, this is not the case in the current context, since $\varvec{\Phi }_{\textrm{g}}$ represents a growth that does not change the elastic properties of the material. Thus, we assume that the constitutive parameters of the body occupying $\Omega _0$ and $\Omega _{\textrm{g}}$ are the same. This assumption requires additionally that the body is homogeneous, i.e., its mechanical properties are the same at each point. This is modeled through an energy function that does not depend explicitly on material points and is the same for both configurations $\Omega _0$ and $\Omega _{\textrm{g}}$, regardless of the growth deformation $\varvec{\Phi }_\textrm{g}$. This stored energy function is denoted by $W_0: \mathbb {R}^{N\times N}_+ \rightarrow \mathbb {R}$, where $\mathbb {R}^{N\times N}_+$ designates the set of square $N\times N$ matrices with strictly positive determinant. The precise assumptions on $W_0$ will be listed in Sect. 2.3.

As is well known, equilibrium configurations $\varvec{\Phi }_{\textrm{e}}$ are minimisers of the functional

2.2 $$\begin{aligned} \int _{\Omega _{\textrm{g}}} W_0 (\varvec{F}_{\textrm{e}}) \, d \varvec{Y} \end{aligned}$$

(to which one may add external forces) over a suitable class of admissible deformations to be specified later. Here $\varvec{F}_{\textrm{e}}$ is the deformation gradient of the elastic deformation $\varvec{\Phi }_{\textrm{e}}$. The variables in $\Omega _{\textrm{g}}$ have been denoted by $\varvec{Y}$, while the variables in $\Omega _0$ by $\varvec{X}$, so that $\varvec{Y} = \varvec{\Phi }_{\textrm{g}} (\varvec{X})$.

Taking into consideration both growth $\varvec{\Phi }_{\textrm{g}}$ and elastic $\varvec{\Phi }_{\textrm{e}}$ deformation, the total deformation $\varvec{\Phi }$ of the body $\Omega _0$ is expressed as the composition of both mappings, i.e., $\varvec{\Phi } = \varvec{\Phi }_{\textrm{e}} \circ \varvec{\Phi }_{\textrm{g}}$. Accordingly, the deformation gradient tensor $\varvec{F}$ associated with $\varvec{\Phi }$ is given by

2.3 $$\begin{aligned} \varvec{F} (\varvec{X}) = \varvec{F}_{\textrm{e}} (\varvec{\Phi }_{\textrm{g}} (\varvec{X})) \varvec{G} (\varvec{X}), \end{aligned}$$

where $\varvec{F}_{\textrm{e}}$ is the deformation gradient of $\varvec{\Phi }_{\textrm{e}}$.

**Fig. 1:**
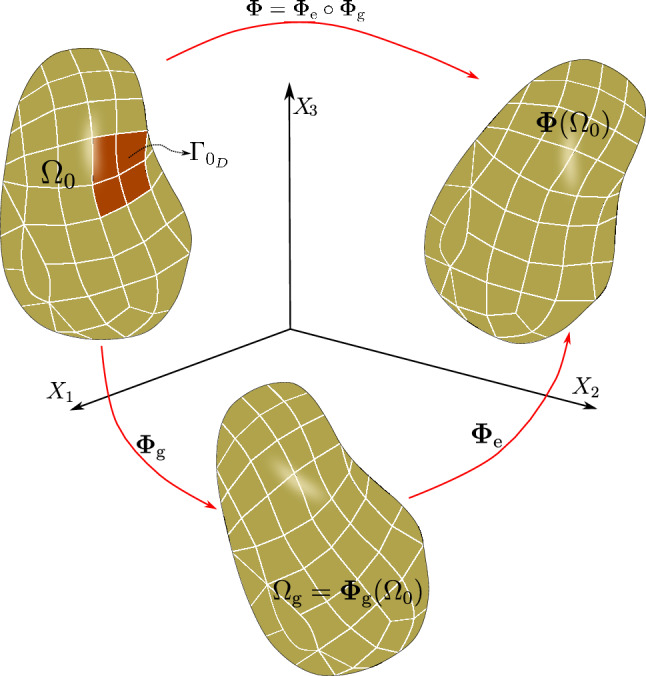
The mapping $\varvec{\Phi }$ between reference $\Omega _0$ and deformed $\Omega$ configurations is expressed as the composition of the growth deformation $\varvec{\Phi }_{\textrm{g}}$ and the elastic deformation $\varvec{\Phi }_{\textrm{e}}$

The three maps involved, $\varvec{\Phi }$, $\varvec{\Phi }_{\textrm{e}}$ and $\varvec{\Phi }_{\textrm{g}}$, are assumed to be orientation-preserving and injective a.e. (see Fig. 1 for a graphical representation).

By (2.1) and the fact that $\varvec{\Phi }_{\textrm{g}}$ is injective a.e., the change of variables $\varvec{Y} = \varvec{\Phi }_{\textrm{g}} (\varvec{X})$ allows rewriting (2.2) in the undeformed configuration $\Omega _0$ as

2.4 $$\begin{aligned} \int _{\Omega _0} W_{\varvec{G}} (\varvec{X}, \varvec{F}) \, d\varvec{X}, \end{aligned}$$

where

2.5 $$\begin{aligned} W_{\varvec{G}} (\varvec{X}, \varvec{F}) := W_0 (\varvec{F} \varvec{G} (\varvec{X})^{-1})\, \det \varvec{G} (\varvec{X}) . \end{aligned}$$

Note that the tensor $\varvec{G}$ breaks the symmetries of $W_0$. Indeed, if $W_0$ has a symmetry group (for example, it is isotropic), then $W_{\varvec{G}}$ does not, in general.

Boundary conditions will be imposed in $\varvec{\Phi }$, but not in $\varvec{\Phi }_{\textrm{g}}$ or $\varvec{\Phi }_{\textrm{e}}$ independently. We will assume that the boundary $\Gamma _0$ of $\Omega _0$ is Lipschitz and is decomposed into two disjoint parts: $\Gamma _{0_D}$ and $\Gamma _{0_N}$, with $\Gamma _{0_D}$ of positive $(N-1)$-dimensional area. On the Dirichlet part $\Gamma _{0_D}$, it is imposed $\varvec{\Phi } = \bar{\varvec{\Phi }}$ for a given deformation $\bar{\varvec{\Phi }}: \Omega _0 \rightarrow \mathbb {R}^N$, while on $\Gamma _{0_N}$ we prescribe the Piola–Kirchhoff stress vector $\varvec{s}_0: \Gamma _{0_N} \rightarrow \mathbb {R}^N$. The latter is not explicitly stated in the admissible set but it is automatically satisfied for minimisers when the surface energy term

2.6 $$\begin{aligned} - \int _{\Gamma _{0_N}} \varvec{s}_0 \cdot \varvec{\Phi } \, d \sigma (\varvec{X}) \end{aligned}$$

is added to the total energy. The cases $\Gamma _{0_N} = \varnothing$ or $\varvec{s}_0 = \varvec{0}$ are not excluded. In fact, volume forces can also be added, whose simplest form is linear:

2.7 $$\begin{aligned} - \int _{\Omega _0} \varvec{f} \cdot \varvec{\Phi } \, d \varvec{X} . \end{aligned}$$

In view of (2.4), the total energy is

2.8 $$\begin{aligned} \Pi _{\varvec{G}} \left( \varvec{\Phi }\right) = \int _{\Omega _0} W_{\varvec{G}} \left( \varvec{X}, \varvec{\nabla } \varvec{\Phi } \right) \, d\varvec{X} - \int _{\Omega _0} \varvec{f} \cdot \varvec{\Phi } \, d \varvec{X} - \int _{\Gamma _{0_N}} \varvec{s}_0 \cdot \varvec{\Phi } \, d \sigma (\varvec{X}) . \end{aligned}$$

Other boundary conditions are also possible (see, e.g., [17, Ch. 5]), as well as more general external forces.

Finally, we fix an exponent $p > 1$ related to the growth at infinity of the function $W_0$ (see Sect. 2.3 for details) and define the class $\mathbb {U}$ of admissible deformations $\varvec{\Phi }$ in (2.8) as

$$\begin{aligned} \mathbb {U}:= \left\{ \varvec{\Phi } \in W^{1,p} (\Omega _0, \mathbb {R}^N) :\varvec{\Phi } = \bar{\varvec{\Phi }} \text { on } \Gamma _{0_D} , \varvec{\Phi } \text { injective, and } \det \varvec{\nabla } \varvec{\Phi }>0 \text { a.e.} \right\} , \end{aligned}$$

where $W^{1,p}$ is the notation for the Sobolev space. Naturally, we suppose that $\mathbb {U}$ is not empty and that $\Pi _{\varvec{G}}$ is not identically infinity in $\mathbb {U}$, which amounts to assuming that $\bar{\varvec{\Phi }} \in \mathbb {U}$ and $\Pi _{\varvec{G}} (\bar{\varvec{\Phi }}) < \infty$.

### Setting of the Shape-Programming Problem

Having in mind potential applications in soft robotics, the so-called shape-programming problem [14] amounts to finding the growth tensor field $\varvec{G}$ in the initial configuration $\Omega _0$ such that the final configuration is as close as possible to a desired target configuration $\Omega _{\textrm{target}}$. Besides reaching this goal, and in order to facilitate its implementation in a possible soft robot, the computed growth tensor field should be as simple as possible.

Inspired by [13], a general form of a complexity functional should have a regularisation term (typically, a squared gradient of $\varvec{G}$) plus a term penalising the difference between the actual $\varvec{G}$ and the target $\varvec{G}_{\textrm{target}}$; such target may well be the identity. These two ingredients should give rise to a simple growth tensor; indeed, the regularising term avoids oscillations, while the penalising term makes $\varvec{G}$ similar to $\varvec{G}_{\textrm{target}}$, which is chosen to be simple, too. In Sect. 2.4 we will describe some possibilities of complexity functions, but for the moment we can think of the functional

$$\begin{aligned} \alpha _1 \int _{\Omega _0}\Vert \varvec{G} - \varvec{G}_{\textrm{target}}\Vert ^2 \, d\varvec{X} + \alpha _2 \int _{\Omega _0}\Vert \varvec{\nabla } \varvec{G} \Vert ^2 \, d\varvec{X}, \end{aligned}$$

for $\alpha _1, \alpha _2 >0$, and develop the mathematical theory for general functionals of the form

$$\begin{aligned} \mathcal {C} (\varvec{G}):= \int _{\Omega _0} \phi ( \varvec{X}, \varvec{G} ) \, d\varvec{X} + \alpha _2 \int _{\Omega _0}\Vert \varvec{\nabla } \varvec{G} \Vert ^2 \, d\varvec{X} \end{aligned}$$

for a certain appropriate density $\phi$. Here above, the norm of a second-order tensor $\varvec{A}$ is defined by $\Vert \varvec{A}\Vert ^2 = \sum _{i, j=1}^N A_{ij}^2$. Similarly is defined the norm of vectors and third-order tensors.

We will see in Sect. 3 that, in order to prove existence of minimisers $\varvec{\Phi }\in \mathbb {U}$ of (2.8), one needs that $\varvec{G}$ be in $L^{\infty }$ and that $\det \varvec{G}$ is bounded away from zero. These facts are in agreement with the requirement that $\varvec{G}$ is easily reachable. The most general way of expressing these assumptions is to fix a compact set $K \subset \mathbb {R}^{N\times N}_+$ and impose that

$$\begin{aligned} \varvec{G} (\varvec{X}) \in K \qquad \text {for a.e. } \varvec{X} \in \Omega _0. \end{aligned}$$

Two relevant examples of the set *K* are

$$\begin{aligned} K = \{ \varvec{F} \in \mathbb {R}^{N\times N}: \Vert \varvec{F} \Vert \le M \text { and } \det \varvec{F} \ge m \} \end{aligned}$$

for some $M, m >0$, and

$$\begin{aligned} K = \{ \varvec{F} \in \mathbb {R}^{N\times N}_+: m_1 \le \sigma ( \varvec{F}) \le m_2 \text { for any singular value } \sigma \text { of } \varvec{F} \} \end{aligned}$$

for some $0 < m_1 \le m_2$. In addition, one may want to model the growth given by $\varvec{G}$ as incompressible; in this case, relevant sets *K* are

$$\begin{aligned} K = \{ \varvec{F} \in \mathbb {R}^{N\times N}: \Vert \varvec{F} \Vert \le M \text { and } \det \varvec{F} = 1 \} \end{aligned}$$

and

$$\begin{aligned} \{ \varvec{F} \in \mathbb {R}^{N\times N}\!: \det \varvec{F} = 1 \text { and } m_1 \le \sigma ( \varvec{F}) \le m_2 \text { for any singular value}\, \sigma \,\text { of}\, \varvec{F} \}. \end{aligned}$$

Concerning the goal that the final configuration is as close as possible to a desired one, there are several options as for the distance between shapes. The simplest, but least realistic, is to consider an $L^2$ distance between the actual and a target deformation; the disadvantage of this choice is that, in general, a distance between deformations is only vaguely related to a distance between shapes. Introduced by [18] and analysed in [8], we consider instead the Hausdorff distance between the image domain and the target set as an adequate way of measuring distances between those sets. As in those works, we use in fact the following smooth approximation of the Hausdorff distance. Let $\Omega (\varvec{G}) = \varvec{\Phi } (\Omega _0)$ be the image domain and $\Omega _{\textrm{target}}$ the target domain. We fix three exponents $\alpha , \beta , \gamma >0$ and a continuous and strictly decreasing function $\varphi : [0, \infty ) \rightarrow (0, \infty )$. We define

$$\begin{aligned}{} & {} \tilde{d}_{\Omega \left( \varvec{G}\right) } \left( \varvec{y}\right) = \varphi ^{-1}\left( \left( \frac{1}{\vert \Omega \left( \varvec{G}\right) \vert } \int _{\Omega \left( \varvec{G}\right) } \varphi ^{\alpha }\left( \Vert \varvec{y}-\varvec{x} \Vert \right) \, d\varvec{x}\right) ^{1/\alpha }\right) , \qquad \varvec{y} \in \Omega _{\textrm{target}},\\{} & {} \quad \tilde{d}_{\Omega _{\textrm{target}}} \left( \varvec{x}\right) = \varphi ^{-1}\left( \left( \frac{1}{\vert \Omega _{\textrm{target}}\vert } \int _{\Omega _{\textrm{target}}}\varphi ^{\alpha }\left( \Vert \varvec{y}-\varvec{x}\Vert \right) \, d\varvec{y}\right) ^{1/\alpha }\right) , \qquad \varvec{x} \in \Omega \left( \varvec{G}\right) ,\\{} & {} \quad \tilde{d}_{\Omega _{\textrm{target}}, \Omega \left( \varvec{G}\right) }:= \left( \frac{1}{\vert \Omega _{\textrm{target}}\vert } \int _{\Omega _{\textrm{target}}} \tilde{d}_{\Omega \left( \varvec{G}\right) } ^{\beta } (\varvec{y}) \, d \varvec{y} \right) ^{1/\beta },\\{} & {} \quad \tilde{d}_{\Omega \left( \varvec{G}\right) , \Omega _{\textrm{target}}}:= \left( \frac{1}{\vert \Omega \left( \varvec{G}\right) \vert } \int _{\Omega \left( \varvec{G}\right) } \tilde{d}_{\Omega _{\textrm{target}}}^{\beta } (\varvec{x}) \, d \varvec{x} \right) ^{1/\beta } \end{aligned}$$

(where $\vert A \vert$ is the volume of the set *A*), so that an approximation of the Hausdorff distance is

2.9 $$\begin{aligned} \rho _H \left( \Omega \left( \varvec{G}\right) ,\Omega _{\textrm{target}}\right) = \left( \tilde{d}_{\Omega _{\textrm{target}}, \Omega \left( \varvec{G}\right) }^{\gamma } + \tilde{d}_{\Omega \left( \varvec{G}\right) ,\Omega _{\textrm{target}}}^{\gamma } \right) ^{1/\gamma }. \end{aligned}$$

Putting all things together, the formulation of the shape-programming problem is:

2.10 $$\begin{aligned} {\left\{ \begin{array}{ll} \text {Minimise in } \varvec{G}: &{} \mathcal {J} \left( \varvec{G} \right) := \rho _H \left( \Omega \left( \varvec{G}\right) ,\Omega _{\textrm{target}}\right) + \mathcal {C} \left( \varvec{G}\right) \\ \text {subject to:} &{} \varvec{G}\in H^1\left( \Omega _0; K \right) \text { is the gradient of an a.e.\ injective map,} \\ &{} \Omega \left( \varvec{G}\right) = \varvec{\Phi }\left( \Omega _0\right) \text {, with } \varvec{\Phi }\in \mathbb {U}\text { a minimiser of } (2.8). \end{array}\right. } \end{aligned}$$

### Choice of the Energy Density

The classical assumptions in nonlinear elasticity for the energy function are *polyconvexity* and *coercivity* [17, 19, 20]. To be precise, we will assume the following conditions for $W_0$:

1. $W_0$ is polyconvex, i.e., there exists a convex function $e: \mathbb {R}^{N\times N}\times \mathbb {R}^{N\times N}\times (0, \infty ) \rightarrow [0,\infty ]$ such that
  2.11 $$\begin{aligned} W_0 (\varvec{F}) = e (\varvec{F}, {\text {cof}}\varvec{F}, \det \varvec{F}) , \qquad \varvec{F} \in \mathbb {R}^{N\times N}_+ . \end{aligned}$$
  If $N=2$, the dependence on ${\text {cof}}\varvec{F}$ can be dispensed with.
2. There exist exponents $p \ge N-1$ and $q \ge \frac{N}{N-1}$ with $p > 1$, and a constant $c>0$ such that
  $$\begin{aligned} W_0(\varvec{F}) \ge c \left( \left\| \varvec{F} \right\| ^p + \left\| {\text {cof}}\varvec{F} \right\| ^q \right) - \frac{1}{c} . \end{aligned}$$
3. $$W_0 (\varvec{F}) \rightarrow \infty $$ as $$\det \varvec{F} \rightarrow 0$$.
4. For every compact $K \subset \mathbb {R}^{N\times N}_+$ there exists $C>0$ such that for all $\varvec{F}_1 \in \mathbb {R}^{N\times N}_+$ and $\varvec{F}_2 \in K$ we have
  $$\begin{aligned} W_0 (\varvec{F}_1 \varvec{F}_2) \le C \left( 1 + W_0 (\varvec{F}_1) \right) . \end{aligned}$$

Of course, ${\text {cof}}$ denotes the cofactor matrix. Conditions (W1)–(W3) are standard [19, 21]. Condition (W2), in fact, implies that any $\varvec{\Phi } \in \mathbb {U}$ with $\Pi _{\varvec{G}} (\varvec{\Phi }) < \infty$ satisfies ${\text {cof}}\varvec{\nabla } \varvec{\Phi } \in L^q (\Omega _0, \mathbb {R}^{N\times N})$ and, thanks to a well-known inequality [21, Eq. (1.4)], $\det \varvec{\nabla } \varvec{\Phi } \in L^{\frac{N q}{N-1}} (\Omega _0)$. Condition (W4) is not standard, but a similar assumption has been used, e.g., in [22]. In the following lemma we show sufficient conditions for the fulfillment of (W4).

#### Lemma 2.1

The following statements hold:

- Let $W_0 \in C ( \mathbb {R}^{N\times N}_+, [0, \infty ))$ be such that there exists $C>0$ for which
  $$\begin{aligned} W_0 \left( \varvec{F}_1 \varvec{F}_2 \right) \le C \left( 1 + W_0 \left( \varvec{F}_1 \right) \right) \left( 1 + W_0 \left( \varvec{F}_2 \right) \right) , \qquad \varvec{F}_1, \varvec{F}_2 \in \mathbb {R}^{N\times N}_+. \end{aligned}$$
  Then $W_0$ satisfies condition (W4).
- For $i=1,2$, let $g_i\in C ( \mathbb {R}^{N\times N}_+, [0, \infty ) )$ and $g_3 \in C ( (0, \infty ), [0, \infty ))$. Assume that there exists $C>0$ such that for all $\varvec{F}_1, \varvec{F}_2 \in \mathbb {R}^{N\times N}_+$ and $t_1, t_2 > 0$,
  $$\begin{aligned} g_i \left( \varvec{F}_1 \varvec{F}_2 \right) \le C \left( 1 + g_i \left( \varvec{F}_1 \right) \right) \left( 1 + g_i \left( \varvec{F}_2 \right) \right) \end{aligned}$$
  and
  $$\begin{aligned} g_3 \left( t_1 t_2 \right) \le C \left( 1 + g_3 \left( t_1 \right) \right) \left( 1 + g_3 \left( t_2 \right) \right) . \end{aligned}$$
  Let $m \in \mathbb {R}$. Then the function
  $$\begin{aligned} W_0 \left( \varvec{F} \right) = g_1 \left( \varvec{F} \right) + g_2 \left( {\text {cof}}\varvec{F} \right) + g_3 \left( \det \varvec{F} \right) + m \end{aligned}$$
  satisfies condition (W4) whenever $W_0 \ge 0$.
- For $i=1,2, 3$, let $h_i \in C ( (0, \infty ), [0, \infty ) )$. Assume that there exists $C>0$ such that for all $t_1, t_2 > 0$,
  2.12 $$\begin{aligned} h_i \left( t_1 t_2 \right) \le C \left( 1 + h_i \left( t_1 \right) \right) \left( 1 + h_i \left( t_2 \right) \right) . \end{aligned}$$
  If $h_1, h_2$ are monotone increasing, and $m \in \mathbb {R}$, then the function
  $$\begin{aligned} W_0 \left( \varvec{F} \right) = h_1 \left( \left\| \varvec{F}\right\| \right) + h_2 \left( \left\| {\text {cof}}\varvec{F} \right\| \right) + h_3 \left( \det \varvec{F} \right) + m \end{aligned}$$
  satisfies condition (W4) whenever $W_0 \ge 0$.
- Let $a, b, c>0$. Then the function
  2.13 $$\begin{aligned} W_0 (\varvec{F}) = a \Vert \varvec{F} \Vert ^2+b\Vert {\text {cof}}\varvec{F} \Vert ^2+c \left( \det \varvec{F} - 1 \right) ^2 - 2(a + 2b) \log (\det \varvec{F}) - 3(a + b) \end{aligned}$$
  satisfies condition (W4).

#### Proof

Part (a). Let $K \subset \mathbb {R}^{N\times N}_+$ be compact. Let $\varvec{F}_1 \in \mathbb {R}^{N\times N}_+$ and $\varvec{F}_2 \in K$. Then

$$\begin{aligned} W_0 \left( \varvec{F}_1 \varvec{F}_2 \right){} & {} \le C \left( 1 + W_0 \left( \varvec{F}_1 \right) \right) \left( 1 + W_0 \left( \varvec{F}_2 \right) \right) \\{} & {} \le C \left( 1 + \left\| W_0 \right\| _{L^{\infty } (K)} \right) \left( 1 + W_0 \left( \varvec{F}_1 \right) \right) . \end{aligned}$$

Part (b). For any $\varvec{F}_1, \varvec{F}_2 \in \mathbb {R}^{N\times N}_+$,

$$\begin{aligned} g_1 \left( \varvec{F}_1 \varvec{F}_2 \right){} & {} \le C \left( 1 + g_1 \left( \varvec{F}_1 \right) \right) \left( 1 + g_1 \left( \varvec{F}_2 \right) \right) \\{} & {} \le C \left( 1 + W_0 \left( \varvec{F}_1 \right) - m \right) \left( 1 + W_0 \left( \varvec{F}_2 \right) - m \right) , \end{aligned}$$

and, analogously,

$$\begin{aligned} g_2 \left( {\text {cof}}(\varvec{F}_1 \varvec{F}_2) \right)&\le C \left( 1 + W_0 \left( \varvec{F}_1 \right) - m \right) \left( 1 + W_0 \left( \varvec{F}_2 \right) - m \right) , \\ g_3 \left( \det (\varvec{F}_1 \varvec{F}_2) \right)&\le C \left( 1 + W_0 \left( \varvec{F}_1 \right) - m \right) \left( 1 + W_0 \left( \varvec{F}_2 \right) - m \right) . \end{aligned}$$

Therefore,

$$\begin{aligned} W_0 \left( \varvec{F}_1 \varvec{F}_2 \right) \le 3 C \left( 1 + W_0 \left( \varvec{F}_1 \right) - m \right) \left( 1 + W_0 \left( \varvec{F}_2 \right) - m \right) + m. \end{aligned}$$

If $$m \ge 0$$ then

$$\begin{aligned}{} & {} W_0 \left( \varvec{F}_1 \varvec{F}_2 \right) \le 3 C \left( 1 + W_0 \left( \varvec{F}_1 \right) \right) \left( 1 + W_0 \left( \varvec{F}_2 \right) \right) + m \\{} & {} \quad \le \left( 3 C + m \right) \left( 1 + W_0 \left( \varvec{F}_1 \right) \right) \left( 1 + W_0 \left( \varvec{F}_2 \right) \right) , \end{aligned}$$

while if $m < 0$ then

$$\begin{aligned} W_0 \left( \varvec{F}_1 \varvec{F}_2 \right)&\le 3 C \left( 1 + W_0 \left( \varvec{F}_1 \right) - m \right) \left( 1 + W_0 \left( \varvec{F}_2 \right) - m \right) \\&\le 3 C \left( 1 + W_0 \left( \varvec{F}_1 \right) \right) \left( 1 + W_0 \left( \varvec{F}_2 \right) \right) , \end{aligned}$$

so $W_0$ satisfies the assumptions of part (a).

Part (c). We define for $i = 1, 2$,

$$\begin{aligned} g_i (\varvec{F}) = h_i \left( \left\| \varvec{F} \right\| \right) , \qquad \varvec{F} \in \mathbb {R}^{N\times N}_+ \end{aligned}$$

and $g_3 = h_3$. Then, for $\varvec{F}_1, \varvec{F}_2 \in \mathbb {R}^{N\times N}_+$ we have

$$\begin{aligned} g_i \left( \varvec{F}_1 \varvec{F}_2 \right) = h_i \left( \left\| \varvec{F}_1 \varvec{F}_2 \right\| \right) \le h_i \left( \left\| \varvec{F}_1 \right\| \left\| \varvec{F}_2 \right\| \right) \le C \left( 1 + g_i \left( \varvec{F}_1 \right) \right) \left( 1 + g_i \left( \varvec{F}_2 \right) \right) . \end{aligned}$$

Thus, $g_1, g_2, g_3$ and *m* satisfy the assumptions of part (b).

Part (d). Define

$$\begin{aligned} h_1 (t){} & {} = a t^2, \quad h_2 (t) = b t^2, \quad h_3 (t) \\{} & {} = c (t-1)^2 - 2 (a+2b) \log t + A \text { and } m = - 3 (a+b) - A, \end{aligned}$$

where $A \in \mathbb {R}$ is chosen so that $h_3 \ge 0$. Then,

$$\begin{aligned} h_1 (t_1 t_2) = a t_1^2 t_2^2 = \frac{1}{a} h_1 (t_1) h_1 (t_2) \le \frac{1}{a} \left( 1 + h(t_1) \right) \left( 1 + h(t_2) \right) . \end{aligned}$$

An analogous bound holds for $h_2$.

As for $h_3$, it is easy to check that there exist $a_1, a_2, b_2, c_1, c_2 > 0$ such that

$$\begin{aligned} {\left\{ \begin{array}{ll} - a_1 \log t \le h_3 (t) \le - a_2 \log t &{} \text {if } 0< t < \frac{1}{2}, \\ h_3 (t) \le b_2 &{} \text {if } \frac{1}{2} \le t \le 2,\\ c_1 t^2 \le h_3 (t) \le c_2 t^2 &{} \text {if } t > 2. \end{array}\right. } \end{aligned}$$

We argue by cases so as to show inequality (2.12) for $h_3$. If $t_1, t_2 < \frac{1}{2}$,

$$\begin{aligned} h_3 (t_1 t_2)&\le - a_2 \log (t_1 t_2) = a_2 \left( - \log t_1 - \log t_2 \right) \le \frac{a_2}{a_1} \left( h_3 (t_1) + h_3 (t_2) \right) \\&\le \frac{a_2}{a_1} \left( 1 + h_3 (t_1) \right) \left( 1 + h_3 (t_2) \right) . \end{aligned}$$

If $$t_1 < \frac{1}{2} \le t_2 \le 2$$ and $$t_1 t_2 < \frac{1}{2}$$,

$$\begin{aligned} h_3 (t_1 t_2)&\le a_2 \left( - \log t_1 - \log t_2 \right) \le a_2 \left( \frac{1}{a_1} h_3 (t_1) + \log 2 \right) \\&\le a_2 \left( \frac{1}{a_1} + \log 2 \right) \left( 1 + h_3 (t_1) \right) \left( 1 + h_3 (t_2) \right) . \end{aligned}$$

If $$\frac{1}{2} \le t_1 t_2 \le 2$$,

$$\begin{aligned} h_3 (t_1 t_2) \le b_2 \le b_2 \left( 1 + h_3 (t_1) \right) \left( 1 + h_3 (t_2) \right) . \end{aligned}$$

If $$t_1< \frac{1}{2}< 2 < t_2$$ and $$t_1 t_2 < \frac{1}{2}$$,

$$\begin{aligned} h_3 (t_1 t_2) \le a_2 \left( - \log t_1 - \log t_2 \right) \le \frac{a_2}{a_1} h_3 (t_1) \le \frac{a_2}{a_1} \left( 1 + h_3 (t_1) \right) \left( 1 + h_3 (t_2) \right) . \end{aligned}$$

If $$t_1< \frac{1}{2}< 2 < t_2$$ and $$t_1 t_2 > 2$$,

$$\begin{aligned} h_3 (t_1 t_2) \le c_2 t_1^2 t_2^2 \le \frac{c_2}{4 c_1} h_3 (t_2) \le \frac{c_2}{4 c_1} \left( 1 + h_3 (t_1) \right) \left( 1 + h_3 (t_2) \right) . \end{aligned}$$

If $$\frac{1}{2} \le t_1, t_2 \le 2$$ and $$t_1 t_2 < \frac{1}{2}$$,

$$\begin{aligned} h_3 (t_1 t_2) \le a_2 \left( - \log t_1 - \log t_2 \right) \le 2 a_2 \log 2 \le 2 a_2 \log 2 \left( 1 + h_3 (t_1) \right) \left( 1 + h_3 (t_2) \right) . \end{aligned}$$

If $$\frac{1}{2} \le t_1, t_2 \le 2$$ and $$t_1 t_2 > 2$$,

$$\begin{aligned} h_3 (t_1 t_2) \le c_2 t_1^2 t_2^2 \le c_2 b_2^4 \le c_2 b_2^4 \left( 1 + h_3 (t_1) \right) \left( 1 + h_3 (t_2) \right) . \end{aligned}$$

If $$\frac{1}{2} \le t_1 \le 2 < t_2$$ and $$t_1 t_2 > 2$$,

$$\begin{aligned} h_3 (t_1 t_2) \le c_2 t_1^2 t_2^2 \le \frac{{{4}} c_2}{c_1} h_3 (t_2) \le \frac{{{4}} c_2}{c_1} \left( 1 + h_3 (t_1) \right) \left( 1 + h_3 (t_2) \right) . \end{aligned}$$

If $$t_1, t_2 > 2$$,

$$\begin{aligned} h_3 (t_1 t_2) \le c_2 t_1^2 t_2^2 \le \frac{c_2}{c_1^2} h_3 (t_1) h_2 (t_2) \le \frac{c_2}{c_1^2} \left( 1 + h_3 (t_1) \right) \left( 1 + h_3 (t_2) \right) . \end{aligned}$$

We have thus shown that $$h_1, h_2, h_3 $$ and *m* satisfy the assumptions of part (c). $$\square $$

Condition (a) of Lemma 2.1 appears in [22, Rk. 2.3]. It turns out that there are many useful examples of energy densities satisfying (W1)–(W4) that are widely used in nonlinear elasticity (see, e.g., [17, 19]). For example, condition (W1) is fulfilled when assumption (c) in Lemma 2.1 holds with $h_i$ convex, while conditions (W2)–(W3) are, in general, easy to verify.

The numerical simulations of Sect. 5 will use the Mooney–Rivlin material in (2.13) when $N=3$. In this section we have shown that this $W_0$ satisfies conditions (W1) and (W4). Condition (W3), on the other hand, is clear, while condition (W2) is easily seen to hold for the exponents $p=q=2$.

### Choices of Complexity Functionals

The work [13] introduces some examples of complexity functionals in the context of different active materials. In the presence of isotropy, their functionals are based on the right Cauchy–Green deformation tensor $\varvec{C}_{\textrm{g}} = \varvec{G}^T \varvec{G}$ associated with growth. However, we have found several advantages to treat $\varvec{G}$, as opposed to $\varvec{C}_{\textrm{g}}$ as the main variable. Indeed, dealing with $\varvec{C}_{\textrm{g}}$ involves the use of the so-called intrinsic elasticity [23, Sect. 4.2] and needs to incorporate the constraint that $\varvec{C}_{\textrm{g}}$ is a metric tensor, which is difficult to handle.

One of the examples presented in [13] is

2.14 $$\begin{aligned} \mathcal {C}_1\left( \varvec{C}_{\textrm{g}}\right) : = \alpha _1 \int _{\Omega _0}\Vert \varvec{C}_{\textrm{g}} - \varvec{C}_{\textrm{target}}\Vert ^2 \, d\varvec{X} + \alpha _2 \int _{\Omega _0}\Vert \varvec{\nabla } \varvec{C}_{\textrm{g}} \Vert ^2 \, d\varvec{X}, \end{aligned}$$

with $\varvec{C}_{\textrm{target}}$ a given target and $\alpha _1, \alpha _2 >0$ weighting parameters. Although not explicitly mentioned in [13], similar in spirit is the functional

2.15 $$\begin{aligned} \mathcal {C}_2 \left( \varvec{C}_{\textrm{g}}\right) : = \alpha _1 \int _{\Omega _0}\left\| \frac{\varvec{C}_{\textrm{g}}}{\left( \det \varvec{C}_{\textrm{g}} \right) ^{1/N}} - \varvec{C}_{\textrm{target}} \right\| ^2 \, d\varvec{X} + \alpha _2 \int _{\Omega _0}\Vert \varvec{\nabla } \varvec{C}_{\textrm{g}} \Vert ^2 \, d\varvec{X}, \end{aligned}$$

with $\det \varvec{C}_{\textrm{target}} = 1$, in which the penalizing term only accounts for the dissimilarity of $\varvec{C}_{\textrm{g}}$ from $\varvec{C}_{\textrm{target}}$ in shape but not in volume. Since in our context, we have decided to work with $\varvec{G}$ as the main variable, the counterparts of $\mathcal {C}_1$ and $\mathcal {C}_2$ are

$$\begin{aligned} \bar{\mathcal {C}}_1\left( \varvec{G}\right) : = \alpha _1 \int _{\Omega _0}{{\,\textrm{dist}\,}}\left( \varvec{G}, SO(N) \varvec{G}_{\textrm{target}} \right) ^2 \, d\varvec{X} + \alpha _2 \int _{\Omega _0}\Vert \varvec{\nabla } \varvec{G} \Vert ^2 \, d\varvec{X}, \end{aligned}$$

and

$$\begin{aligned} \bar{\mathcal {C}}_2 \left( \varvec{G}\right) : = \alpha _1 \int _{\Omega _0} {{\,\textrm{dist}\,}}\left( \frac{\varvec{G}}{\left( \det \varvec{G} \right) ^{1/N}}, SO(N) \varvec{G}_{\textrm{target}} \right) ^2 \, d\varvec{X} + \alpha _2 \int _{\Omega _0}\Vert \varvec{\nabla } \varvec{G} \Vert ^2 \, d\varvec{X}, \end{aligned}$$

respectively, for a given target $\varvec{G}_{\textrm{target}}$, which in $\bar{\mathcal {C}}_2$ satisfies $\det \varvec{G}_{\textrm{target}} = 1$. Here *SO*(*N*) denotes the set of (proper) rotations, and ${{\,\textrm{dist}\,}}$ the distance between a matrix a set of matrices, i.e., the minimum distance between the matrix and any element of the set of matrices. Note that in $\bar{\mathcal {C}}_1$ we wrote ${{\,\textrm{dist}\,}}(\varvec{G}, SO(N) \varvec{G}_{\textrm{target}})$ instead of $\Vert \varvec{G} - \varvec{G}_{\textrm{target}} \Vert$ to guarantee frame-indifference and isotropy. Analogously for $\bar{\mathcal {C}}_2$.

A final comment refers to the regularising term with integrand $\Vert \varvec{\nabla } \varvec{G} \Vert ^2$ that is to be used in any complexity functional involved. As a matter of fact, from a practical point of view, it may be advantageous to substitute it by a standard regularising Helmholtz filter $\hat{\varvec{G}}$ of the form

2.16 $$\begin{aligned} {\left\{ \begin{array}{ll} \hat{\varvec{G}} - l^2 \varvec{\Delta } \hat{\varvec{G}} =\varvec{G} &{} \text {in}\,\,\Omega _0,\\ \varvec{\nabla } \hat{\varvec{G}}\cdot \varvec{N}=\textbf{0} &{} \text {on} \,\,\partial \Omega _0. \end{array}\right. } \end{aligned}$$

Here $l>0$ acts as a length-scale parameter controlling the amplitude of the regularisation, $\varvec{\Delta }$ denotes the Laplacian operator, and $\varvec{N}$ stands for the outer unit normal vector to $\partial \Omega _0$. In this case, we replace the term

$$\begin{aligned} \int _{\Omega _0}\Vert \varvec{\nabla } \varvec{G} \Vert ^2 \, d\varvec{X} \end{aligned}$$

by the $L^2$-norm of $\hat{\varvec{G}}$. Note that the operation $G\mapsto {\tilde{G}}$ enjoys much better analytical properties than $G\mapsto \nabla G$: the former is a compact operation with nice properties even from the approximation perspective, while the latter is not even continuous. In addition, as just remarked, parameter *l* can be directly associated with the length-scale of the regularization, a feature that is very convenient form the practical viewpoint. At any rate, this filter has performed quite well in the simulations below. In fact, a different version of the Helmholtz filter more suitable for the implementation will be finally adopted in the numerical simulations. We will explain later how to adapt the proof of existence to these cases.

## Mathematical Analysis

This section aims at providing a rigorous mathematical analysis of the shape programming problem (2.10). We shall proceed in two steps. We will first prove that for a given growth tensor $\varvec{G}$, there exist minimisers of (2.8). Then, existence of solutions for (2.10) is established.

The following lemma is an easy consequence of formula

$$\begin{aligned} \varvec{A}^{-1} = \frac{{\text {cof}}\varvec{A}^T}{\det \varvec{A}}, \qquad \varvec{A} \in \mathbb {R}^{N\times N}_+. \end{aligned}$$

### Lemma 3.1

Let $K \subset \mathbb {R}^{N\times N}_+$ be compact. Then there exist compact sets $K_1 \subset \mathbb {R}^{N\times N}_+$ and $K_2 \subset (0, \infty )$ such that for all $\varvec{G} \in L^{\infty } (\Omega _0, K)$ we have ${\text {cof}}\varvec{G}, \varvec{G}^{-1}, {\text {cof}}\varvec{G}^{-1} \in L^{\infty } (\Omega _0, K_1)$ and $\det \varvec{G}, \det \varvec{G}^{-1} \in L^{\infty } (\Omega _0, K_2)$. Moreover, if $\{\varvec{G}_j\}_{j \in \mathbb {N}}$ is a sequence in $L^{\infty } (\Omega _0, K)$ such that

$$\begin{aligned} \varvec{G}_j\rightarrow \varvec{G} \qquad \text {a.e.} \end{aligned}$$

then

3.1 $$\begin{aligned} {\text {cof}}\varvec{G}_j \rightarrow {\text {cof}}\varvec{G} \quad \text {and} \quad \det \varvec{G}_j \rightarrow \det \varvec{G} \qquad \text {a.e.} \end{aligned}$$

and

3.2 $$\begin{aligned} \varvec{G}_j^{-1} \rightarrow \varvec{G}^{-1} , \qquad {\text {cof}}\varvec{G}_j^{-1} \rightarrow {\text {cof}}\varvec{G}^{-1} \quad \text {and} \quad \det \varvec{G}_j^{-1} \rightarrow \det \varvec{G}^{-1} \qquad \text {a.e.} \end{aligned}$$

The following lower semicontinuity result will help in the final steps of the main proof. Recall that for each measurable $\varvec{G}: \Omega _0 \rightarrow \mathbb {R}^{N\times N}_+$, the function $W_{\varvec{G}}: \Omega _0 \times \mathbb {R}^{N\times N}_+ \rightarrow \mathbb {R}\cup \{ \infty \}$ is defined as in (2.5).

### Lemma 3.2

Let $W_0: \mathbb {R}^{N\times N}_+ \rightarrow \mathbb {R}\cup \{ \infty \}$ satisfy conditions (W1)–(W3) of Sect. 2.3. Let $\{\varvec{\Phi }_j\}_{j \in \mathbb {N}}$ be a sequence in $\mathbb {U}$ such that

$$\begin{aligned} \varvec{\Phi }_j\rightharpoonup \varvec{\Phi },\quad {\text {cof}}\varvec{\nabla } \varvec{\Phi }_j\rightharpoonup {\text {cof}}\varvec{\nabla } \varvec{\Phi },\quad \det \varvec{\nabla } \varvec{\Phi }_j\rightharpoonup \det \varvec{\nabla } \varvec{\Phi } \qquad \text {in } L^1 (\Omega _0). \end{aligned}$$

Let $K \subset \mathbb {R}^{N\times N}_+$ be compact and let $\{\varvec{G}_j\}_{j \in \mathbb {N}}$ be a sequence in $L^\infty (\Omega _0, K)$ such that

$$\begin{aligned} \varvec{G}_j\rightarrow \varvec{G} \qquad \text {a.e.} \end{aligned}$$

Then

$$\begin{aligned} \int _{\Omega _0} W_{\varvec{G}} (\varvec{X}, \varvec{\nabla } \varvec{\Phi }) \,d\varvec{X} \le \liminf _{j\rightarrow \infty } \int _{\Omega _0} W_{\varvec{G}_j} (\varvec{X}, \varvec{\nabla } \varvec{\Phi }_j) \,d\varvec{X}. \end{aligned}$$

### Proof

Lemma 3.1 yields convegences (3.1) and (3.2). By a standard fact on the product of two sequences, one factor converging weakly in $L^1$ and the other one a.e. with and $L^{\infty }$ bound (see, e.g., [24, Prop. 2.61]), we obtain thanks to Lemma 3.1 that

$$\begin{aligned}&\varvec{\nabla } \varvec{\Phi }_j \varvec{G}_j^{-1} \rightharpoonup \varvec{\nabla } \varvec{\Phi } \varvec{G}^{-1} , \qquad {\text {cof}}\varvec{\nabla } \varvec{\Phi }_j {\text {cof}}\varvec{G}_j^{-1} \rightharpoonup {\text {cof}}\varvec{\nabla } \varvec{\Phi } {\text {cof}}\varvec{G}^{-1} , \\&\quad \det \varvec{\nabla } \varvec{\Phi }_j \det \varvec{G}_j^{-1} \rightharpoonup \det \varvec{\nabla } \varvec{\Phi } \det \varvec{G}^{-1} \qquad \text {in } L^1 (\Omega _0). \end{aligned}$$

To sum up, we have the convergences

$$\begin{aligned}&\varvec{\nabla } \varvec{\Phi }_j \varvec{G}_j ^{-1}\rightharpoonup \varvec{\nabla } \varvec{\Phi } \varvec{G}^{-1} , \qquad {\text {cof}}\left( \varvec{\nabla } \varvec{\Phi }_j \varvec{G}_j^{-1} \right) \rightharpoonup {\text {cof}}\left( \varvec{\nabla } \varvec{\Phi } \varvec{G}^{-1} \right) , \\&\quad \det \left( \varvec{\nabla } \varvec{\Phi }_j \varvec{G}_j^{-1} \right) \rightharpoonup \det \left( \varvec{\nabla } \varvec{\Phi } \varvec{G}^{-1}\right) \qquad \text {in } L^1 (\Omega _0) , \end{aligned}$$

as well as $\det \varvec{G}_j \rightarrow \det \varvec{G}$ a.e. with and $L^{\infty }$ bound, which allows us to apply a standard lower semicontinuity result for polyconvex functions (see, e.g., [25, Th. 5.4] or [24, Cor. 7.9]) and conclude that

$$\begin{aligned} \int _{\Omega _0} W_0 (\varvec{X}, \varvec{\nabla } \varvec{\Phi } \varvec{G}^{-1} ) \det \varvec{G} \,d\varvec{X} \le \liminf _{j\rightarrow \infty } \int _{\Omega _0} W_0 (\varvec{X}, \varvec{\nabla } \varvec{\Phi }_j \varvec{G}_j^{-1}) \det \varvec{G}_j \,d\varvec{X}. \end{aligned}$$

This proves the result. $\square$

### Existence of $\varvec{\Phi }$ Given $\varvec{G}$

Before presenting the existence theorems, we recall a property stating that the limit of injective a.e. functions is injective a.e.

#### Proposition 3.3

Let $p \ge N-1$ and $q \ge \frac{N}{N-1}$. For each $j \in \mathbb {N}$, let $\varvec{\Phi }_j, \varvec{\Phi } \in W^{1,p} (\Omega _0, \mathbb {R}^N)$ satisfy $\varvec{\Phi }_j \rightarrow \varvec{\Phi }$ a.e., $\det \varvec{\nabla } \varvec{\Phi }_j \rightharpoonup \det \varvec{\nabla } \varvec{\Phi }$ in $L^1 (\Omega _0)$ and the sequence $\{ {\text {cof}}\varvec{\nabla } \varvec{\Phi }_j\}_{j \in \mathbb {N}}$ is bounded in $L^1 (\Omega _0, \mathbb {R}^{N\times N})$. Assume that ${\text {cof}}\varvec{\nabla } \varvec{\Phi }_j \in L^q (\Omega _0, \mathbb {R}^{N\times N})$, $\varvec{\Phi }_j$ is injective a.e. with $\det \varvec{\nabla } \varvec{\Phi }_j > 0$ a.e. for each $j \in \mathbb {N}$, and $\det \varvec{\nabla } \varvec{\Phi } > 0$ a.e. Then $\varvec{\Phi }$ is injective a.e.

#### Proof

Since $p \ge N-1$ and $q \ge \frac{N}{N-1}$, by [21, Th. 3.2] (see also [16, Prop. 3]), the surface energy $\bar{\mathcal {E}}$ defined in [16, Def. 2] satisfies $\bar{\mathcal {E}} (\varvec{\Phi }_j) = 0$ for each $j \in \mathbb {N}$. The fact that $\varvec{\Phi }_j$ is injective a.e. for each $j \in \mathbb {N}$ lets us conclude ( [16, Th. 2]) that $\varvec{\Phi }$ is injective a.e. $\square$

The following fundamental existence theorem in nonlinear elasticity will be used throughout. Its proof is the sum of deep and fundamental results in Analysis that are indicated below.

#### Theorem 3.4

Assume that $W: \Omega _0 \times \mathbb {R}^{N\times N}_+ \rightarrow [0, \infty ]$ satisfies the following conditions:

- *W* is $\mathcal {L} \times \mathcal {B}$-measurable, where $\mathcal {L}$ denotes the Lebesgue $\sigma$-algebra in $\Omega _0$, and $\mathcal {B}$ stands for the Borel $\sigma$-algebra in $\mathbb {R}^{N\times N}$.
- $W (\varvec{X}, \cdot )$ is polyconvex for a.e. $\varvec{X} \in \Omega _0$.
- There exist exponents $p \ge N-1$ and $q \ge \frac{N}{N-1}$ with $p > 1$, and a constant $c>0$ such that
  $$\begin{aligned} W(\varvec{X}, \varvec{F}) \ge c \left( \Vert \varvec{F}\Vert ^p + \Vert {\text {cof}}\varvec{F}\Vert ^q \right) - \frac{1}{c}, \qquad \text {for a.e. } \varvec{X} \in \Omega _0 \text { and all } \varvec{F} \in \mathbb {R}^{N\times N}_+. \end{aligned}$$
- $$W (\varvec{X}, \varvec{F}) \rightarrow \infty $$ as $$\det \varvec{F} \rightarrow 0$$, for a.e. $$\varvec{X} \in \Omega _0$$.

Assume that $\Gamma _{0_D}$ is an $(N-1)$-rectifiable subset of $\partial \Omega$ of positive $(N-1)$-dimensional measure and that $\bar{\varvec{\Phi }}: \Gamma _{0_D} \rightarrow \mathbb {R}^N$ is measurable. Let $\varvec{f} \in L^2 (\Omega _0, \mathbb {R}^N)$ and $\varvec{s}_0 \in L^2 (\Gamma _{0_N}, \mathbb {R}^N)$.

Let the functional

$$\begin{aligned} I(\varvec{\Phi }):= \int _{\Omega _0} W (\varvec{X}, \varvec{\nabla } \varvec{\Phi } (\varvec{X})) \, d \varvec{X} - \int _{\Omega _0} \varvec{f} \cdot \varvec{\Phi } \, d \varvec{X} - \int _{\Gamma _{0_N}} \varvec{s}_0 \cdot \varvec{\Phi } \, d \sigma (\varvec{X}). \end{aligned}$$

be defined in $\mathbb {U}$. Assume that $\mathbb {U}\ne \varnothing$ and that *I* is not identically infinity in $\mathbb {U}$. Then there exists a minimiser of *I* in $\mathbb {U}$.

#### Proof

The treatment of the linear terms (2.6) and (2.7) is standard (e.g., [19, Sect. 7] or [17, Ch. 5]), so we can assume $\varvec{f} = \varvec{0}$ and $\varvec{s}_0 = \varvec{0}$.

Since $\mathbb {U}\ne \varnothing$ and *I* is not identically infinity in $\mathbb {U}$, there exists a minimising sequence $\{ \varvec{\Phi }_j \}_{j \in \mathbb {N}}$ of *I* in $\mathbb {U}$. Thus, $\{ I ( \varvec{\Phi }_j ) \}_{j \in \mathbb {N}}$ is bounded and, by condition (c), we have that $\{ \varvec{\nabla } \varvec{\Phi }_j \}_{j \in \mathbb {N}}$ is bounded in $L^p$ and $\{ {\text {cof}}\varvec{\nabla } \varvec{\Phi }_j \}_{j \in \mathbb {N}}$ is bounded in $L^q$. Poincaré’s inequality shows that $\{ \varvec{\Phi }_j \}_{j \in \mathbb {N}}$ is bounded in $W^{1,p}$. Since $p>1$, there exist $\varvec{\Phi } \in L^p$ and a subsequence (not relabelled) such that $\varvec{\Phi }_j \rightharpoonup \varvec{\Phi }$ in $W^{1,p}$. The continuity of traces shows that $\varvec{\Phi }$ satisfies the boundary condition. By [21, Lemma 4.1],

$$\begin{aligned} {\text {cof}}\varvec{\nabla } \varvec{\Phi }_j \rightharpoonup {\text {cof}}\varvec{\nabla } \varvec{\Phi }\text { in }L^q(F), \quad \text {and} \quad \det \varvec{\nabla } \varvec{\Phi }_j \rightharpoonup \det \varvec{\nabla } \varvec{\Phi }\text { in }L^1 (F) \end{aligned}$$

for any compact $F \subset \Omega _0$, as $j \rightarrow \infty$. By the lower semicontinuity of polyconvex functionals (see, e.g., [25, Th. 5.4]),

$$\begin{aligned} \int _F W (\varvec{X}, \varvec{\nabla } \varvec{\Phi } (\varvec{X})) \, d \varvec{X} \le \liminf _{j \rightarrow \infty } \int _F W (\varvec{X}, \varvec{\nabla } \varvec{\Phi }_j (\varvec{X})) \, d \varvec{X} \le \liminf _{j \rightarrow \infty } I (\varvec{\Phi }_j). \end{aligned}$$

Since this is true for all compact $F \subset \Omega _0$, by monotone convergence, we obtain

$$\begin{aligned} I (\varvec{\Phi }) \le \liminf _{j \rightarrow \infty } I (\varvec{\Phi }_j). \end{aligned}$$

Now we show that $\det \varvec{\nabla } \varvec{\Phi } > 0$ a.e. Since $\det \varvec{\nabla } \varvec{\Phi }_j \rightharpoonup \det \varvec{\nabla } \varvec{\Phi }$ in $L^1$ and $\det \varvec{\nabla } \varvec{\Phi }_j > 0$ a.e. for all $j \in \mathbb {N}$, we have that $\det \varvec{\nabla } \varvec{\Phi } \ge 0$ a.e. Let *A* be the set of $\varvec{X} \in \Omega$ such that $\det \varvec{\nabla } \varvec{\Phi } (\varvec{X}) = 0$. We have that $\det \varvec{\nabla } \varvec{\Phi }_j \rightarrow 0$ a.e. in *A*. If $\vert A \vert >0$, by Fatou’s lemma and (d),

$$\begin{aligned} \infty \le \liminf _{j \rightarrow \infty } \int _A W ( \varvec{X}, \varvec{\nabla } \varvec{\Phi }_j (\varvec{X})) \, d \varvec{X} \le \liminf _{j \rightarrow \infty } I (\varvec{\Phi }_j), \end{aligned}$$

which is a contradiction. Therefore, $\vert A \vert = 0$ and $\det \varvec{\nabla } \varvec{\Phi } > 0$ a.e. By Proposition 3.3, $\varvec{\Phi }$ is injective a.e. Therefore, $\varvec{\Phi } \in \mathbb {U}$, and it is a minimiser of *I* in $\mathbb {U}$. $\square$

Note that the integrability assumptions on $\varvec{f}$ and $\varvec{s}_0$ can be weakened; see, e.g., [19, Sect. 7] or [17, Ch. 5].

In the following result we show how the properties of $W_0$ are transferred to $W_{\varvec{G}}$.

#### Lemma 3.5

Let $W_0: \mathbb {R}^{N\times N}_+ \rightarrow [0, \infty ]$ satisfy conditions (W1)–(W3) of Sect. 2.3. Let $\varvec{G}: \Omega _0 \rightarrow \mathbb {R}^{N\times N}_+$ be measurable. Then:

- $$W_{\varvec{G}}$$ is $$\mathcal {L} \times \mathcal {B}$$-measurable.
- $W_{\varvec{G}} (\varvec{X}, \cdot )$ is polyconvex for all $\varvec{X}\in \Omega _0$.
- Let $K \subset \mathbb {R}^{N\times N}_+$ be compact. Then there exists $c_1 >0$ (depending on *K* but not on $\varvec{G}$) such that for any $\varvec{G} \in L^{\infty } (\Omega _0, K)$,
  $$\begin{aligned}{} & {} W_{\varvec{G}} (\varvec{X}, \varvec{F}) \ge c_1 \left( \left\| \varvec{F} \right\| ^p + \left\| {\text {cof}}\varvec{F} \right\| ^q \right) - \frac{1}{c_1}, \\{} & {} \qquad \text {for a.e.\ } \varvec{X} \in \Omega _0 \text { and all } \varvec{F} \in \mathbb {R}^{N\times N}_+. \end{aligned}$$
- $$W_{\varvec{G}} (\varvec{X}, \varvec{F}) \rightarrow \infty $$ as $$\det \varvec{F} \rightarrow 0$$, for a.e. $$\varvec{X} \in \Omega _0$$.

#### Proof

We start by proving (a). As $\varvec{G}$ is measurable, there exists a Borel function $\bar{\varvec{G}}: \Omega _0 \rightarrow \mathbb {R}^{N\times N}_+$ such that $\bar{\varvec{G}} = \varvec{G}$ a.e. Then the function $\varvec{X} \mapsto \bar{\varvec{G}} (\varvec{X})^{-1}$ is Borel in $\Omega _0$ and the function $(\varvec{X}, \varvec{F}) \mapsto \varvec{F} \bar{\varvec{G}} (\varvec{X})^{-1}$ is Borel in $\Omega _0 \times \mathbb {R}^{N\times N}_+$. As $W_0: \mathbb {R}^{N\times N}_+ \rightarrow \mathbb {R}\cup \{\infty \}$ is polyconvex, it is locally Lipschitz in the open set $\{ \varvec{F} \in \mathbb {R}^{N\times N}_+: W_0 (\varvec{F}) < \infty \}$ (see, e.g., [20, Th. 5.3(iv)]), hence Borel in $\mathbb {R}^{N\times N}_+$. Thus, the function $(\varvec{X}, \varvec{F}) \mapsto W_0 (\varvec{F} \bar{\varvec{G}} (\varvec{X})^{-1})$ is Borel in $\Omega _0 \times \mathbb {R}^{N\times N}_+$, and so is the function $(\varvec{X}, \varvec{F}) \mapsto W_0 (\varvec{F} \bar{\varvec{G}} (\varvec{X})^{-1}) \det \bar{\varvec{G}} (\varvec{X})$. Therefore, the function $(\varvec{X}, \varvec{F}) \mapsto W_0 (\varvec{F} \varvec{G} (\varvec{X})^{-1}) \det \varvec{G} (\varvec{X})$ is $\mathcal {L} \times \mathcal {B}$-measurable.

Now we show (b). By definition of polyconvexity, there exists a convex function

$$\begin{aligned} e: \mathbb {R}^{N\times N}\times \mathbb {R}^{N\times N}\times (0, \infty ) \rightarrow \mathbb {R}\cup \{\infty \} \end{aligned}$$

such that (2.11) holds, so

$$\begin{aligned} W_{\varvec{G}} (\varvec{X}, \varvec{F}) = e \left( \varvec{F} \varvec{G} (\varvec{X})^{-1}, {\text {cof}}\varvec{F} {\text {cof}}\varvec{G} (\varvec{X})^{-1}, \det \varvec{F} \det \varvec{G} (\varvec{X})^{-1} \right) \det \varvec{G} (\varvec{X}). \end{aligned}$$

Fix $\varvec{X} \in \Omega _0$. Since *e* is convex, so is the function $\bar{e}: \mathbb {R}^{N\times N}\times \mathbb {R}^{N\times N}\times (0, \infty ) \rightarrow \mathbb {R}\cup \{\infty \}$ given by

$$\begin{aligned} \bar{e} (\varvec{F}, \varvec{H}, J):= e \left( \varvec{F} \varvec{G} (\varvec{X})^{-1}, \varvec{H} {\text {cof}}\varvec{G} (\varvec{X})^{-1}, J \, \det \varvec{G} (\varvec{X})^{-1} \right) \det \varvec{G} (\varvec{X}), \end{aligned}$$

as a composition of a linear map with a convex function. Therefore, the function $W_{\varvec{G}} (\varvec{X}, \cdot )$ is polyconvex.

As for (c), by Lemma 3.1 and using elementary properties of the algebra of square matrices, for a.e. $\varvec{X} \in \Omega _0$,

$$\begin{aligned}&\left\| \varvec{F} \varvec{G} (\varvec{X})^{-1} \right\| \ge \left\| \varvec{G} (\varvec{X}) \right\| ^{-1} \left\| \varvec{F} \right\| \ge \left\| \varvec{G} \right\| _{L^{\infty }}^{-1} \left\| \varvec{F} \right\| , \\&\left\| {\text {cof}}\left( \varvec{F} \varvec{G} (\varvec{X})^{-1} \right) \right\| \ge \left\| {\text {cof}}\varvec{G} (\varvec{X}) \right\| ^{-1} \left\| {\text {cof}}\varvec{F} \right\| \ge \left\| {\text {cof}}\varvec{G} \right\| _{L^{\infty }}^{-1} \left\| {\text {cof}}\varvec{F} \right\| . \end{aligned}$$

Therefore, there exists $c' > 0$ such that

$$\begin{aligned} \left\| \varvec{F} \varvec{G} (\varvec{X})^{-1} \right\| ^p \ge c' \left\| \varvec{F} \right\| ^p, \quad \left\| {\text {cof}}\left( \varvec{F} \varvec{G} (\varvec{X})^{-1} \right) \right\| ^q \ge c' \left\| {\text {cof}}\varvec{F} \right\| ^q, \qquad \varvec{F} \in \mathbb {R}^{N\times N}_+, \end{aligned}$$

which implies (c).

Property (d) is immediate. $\square$

The existence of minimisers of (2.8) for each given, feasible $\varvec{G}$ is now a straightforward consequence of Theorem 3.4 and Lemma 3.5.

#### Theorem 3.6

Let $W_0: \mathbb {R}^{N\times N}_+ \rightarrow [0, \infty ]$ satisfy conditions (W1)–(W3) of Sect. 2.3. Let $K \subset \mathbb {R}^{N\times N}_+$ be compact and $\varvec{G} \in L^{\infty } (\Omega _0, K)$. Assume that $\Gamma _{0_D}$ is an $(N-1)$-rectifiable subset of $\partial \Omega$ of positive $(N-1)$-dimensional measure and that $\bar{\varvec{\Phi }}: \Gamma _{0_D} \rightarrow \mathbb {R}^N$ is measurable. Let $\varvec{f} \in L^2 (\Omega _0, \mathbb {R}^N)$ and $\varvec{s}_0 \in L^2 (\Gamma _{0_N}, \mathbb {R}^N)$. Assume that $\mathbb {U}\ne \varnothing$ and that $\Pi _{\varvec{G}}$ is not identically infinity in $\mathbb {U}$. Then there exists a minimiser of $\Pi _{\varvec{G}}$ in $\mathbb {U}$.

An important issue, which we overlook here, is the potential non-uniqueness of minimiser $\varvec{\Phi }$ for given $\varvec{G}$. A much more delicate analysis would be required to deal with potential bifurcation problems as the tensor $\varvec{G}$ moves in the iterative, approximation procedure implemented in Sect. 5 seeking an optimal $\varvec{G}$. However, if one sticks to a selected continuous branch of solutions, one would end up with an optimal tensor $\varvec{G}$. We have to report no difficulties here in the numerical approximations performed.

### Existence of Optimal $\varvec{G}$

The lower semicontinuity of the function $\rho _H$, as given by (2.9), was shown in [8]. Although the framework here is somewhat different, the same proof is valid. For the convenience of the reader, we state in a precise way the result inside the proof of [8, Th. 4.2] that will be used in Theorem 3.9 below.

#### Proposition 3.7

Let $\{ \varvec{\Phi }_j \}_{j \in \mathbb {N}}$ be a sequence in $W^{1,p} (\Omega _0, \mathbb {R}^N)$ such that

$$\begin{aligned} {\text {cof}}\varvec{\nabla } \varvec{\Phi }_j \in L^{\frac{N}{N-1}} (\Omega _0, \mathbb {R}^{N\times N}),\quad \det \varvec{\nabla } \varvec{\Phi }_j > 0 {{\text { a.e.}}}, \end{aligned}$$

and $\varvec{\Phi }_j$ is injective a.e., for each $j \in \mathbb {N}$. Assume that there exists $\varvec{\Phi } \in W^{1,p} (\Omega _0, \mathbb {R}^N)$, with $\det \varvec{\nabla } \varvec{\Phi } > 0$ a.e., such that

$$\begin{aligned} \varvec{\Phi }_j \rightarrow \varvec{\Phi } \text { a.e.}, \quad \det \varvec{\nabla } \varvec{\Phi }_j \rightharpoonup \det \varvec{\nabla } \varvec{\Phi } \text { in } L^1 (\Omega _0), \quad \sup _{j \in \mathbb {N}} \left\| {\text {cof}}\varvec{\nabla } \varvec{\Phi }_j \right\| _{L^1 (\Omega _0, \mathbb {R}^{N\times N})} < \infty . \end{aligned}$$

Then

$$\begin{aligned} \rho _H \left( \varvec{\Phi } (\Omega ),\Omega _{\textrm{target}}\right) \le \liminf _{j \rightarrow \infty }\rho _H \left( \varvec{\Phi }_j (\Omega ),\Omega _{\textrm{target}}\right) . \end{aligned}$$

The following result is an easy consequence of (W4).

#### Lemma 3.8

Let $W_0: \mathbb {R}^{N\times N}_+ \rightarrow \mathbb {R}\cup \{ \infty \}$ satisfy condition (W4) of Sect. 2.3. Let $K \subset \mathbb {R}^{N\times N}_+$ be compact. Let $\varvec{f} \in L^2 (\Omega _0, \mathbb {R}^N)$ and $\varvec{s}_0 \in L^2 (\Gamma _{0_N}, \mathbb {R}^N)$. Let $\varvec{\Phi } \in \mathbb {U}$. If $\Pi _{\varvec{G}} (\varvec{\Phi }) < \infty$ for some $\varvec{G} \in L^{\infty } (\Omega , K)$ then $\Pi _{\varvec{G}} (\varvec{\Phi }) < \infty$ for all $\varvec{G} \in L^{\infty } (\Omega , K)$.

#### Proof

Let $\varvec{G} \in L^{\infty } (\Omega , K)$, $\varvec{F} \in \mathbb {R}^{N\times N}_+$ and $\varvec{X} \in \Omega _0$. Condition (W4) and the fact $\varvec{G} \in L^{\infty } (\Omega , K)$ imply that

3.3 $$\begin{aligned} W_{\varvec{G}} (\varvec{X}, \varvec{F}) = W_0 (\varvec{F} \varvec{G} (\varvec{X})^{-1}) \det \varvec{G} (\varvec{X}) \le C \left( 1 + W_0 (\varvec{F}) \right) , \end{aligned}$$

for some constant $C>0$. Similarly,

$$\begin{aligned} W_0 (\varvec{F}) \le c_1 \left( 1 + W_0 (\varvec{F} \varvec{G} (\varvec{X})^{-1}) ) \right) = c_1 \left( 1 + \frac{ W_{\varvec{G}} (\varvec{X}, \varvec{F}) }{\det \varvec{G}} \right) \le c_2 \left( 1 + W_{\varvec{G}} (\varvec{X}, \varvec{F}) \right) , \end{aligned}$$

for some constants $c_1, c_2>0$. The conclusion readily follows. $\square$

Our main result is concerned with the existence of an optimal $\varvec{G}$.

#### Theorem 3.9

Let $W_0: \mathbb {R}^{N\times N}_+ \rightarrow [0, \infty ]$ satisfy conditions (W1)–(W4) of Sect. 2.3. Let $K \subset \mathbb {R}^{N\times N}_+$ be compact. Let $\varvec{f} \in L^2 (\Omega _0, \mathbb {R}^N)$ and $\varvec{s}_0 \in L^2 (\Gamma _{0_N}, \mathbb {R}^N)$. Let $\phi : \Omega _0 \times \mathbb {R}^{N\times N}\rightarrow [0, \infty ]$ satisfy:

- $$\phi $$ is $$\mathcal {L} \times \mathcal {B}$$-measurable.
- $\phi (\varvec{X}, \cdot )$ is lower semicontinuous for a.e. $\varvec{X} \in \Omega _0$.

Assume that $\mathbb {U}\ne \varnothing$. Let $\alpha >0$. Define

$$\begin{aligned} \mathcal {J} (\varvec{G}) = \rho _H \left( \Omega \left( \varvec{G}\right) ,\Omega _{\textrm{target}}\right) + \int _{\Omega _0} \left( \alpha \Vert \varvec{\nabla } \varvec{G} (\varvec{X}) \Vert ^2 + \phi (\varvec{X}, \varvec{G} (\varvec{X})) \right) d \varvec{X} \end{aligned}$$

in

$$\begin{aligned} \mathcal {A}= & {} \{ \varvec{G}\in H^1\left( \Omega _0; K\right) \text { is the gradient of an a.e.\ injective map, } \Pi _{\varvec{G}} \text { is not} \\{} & {} \text {identically infinity in }\mathbb {U}, \ \Omega \left( \varvec{G}\right) = \varvec{\Phi }\left( \Omega _0\right) , \text { with } \varvec{\Phi } \text { a minimiser of } \Pi _{\varvec{G}} \text { in } \mathbb {U}\}. \end{aligned}$$

Assume that $\mathcal {A} \ne \varnothing$ and that $\mathcal {J}$ is not identically infinity in $\mathcal {A}$. Then there exists a minimiser of $\mathcal {J}$ in $\mathcal {A}$.

#### Proof

We will rely on the Direct Method of Calculus of Variations. Let $\{ \varvec{G}_j \}_{j \in \mathbb {N}}$ be a minimising sequence of $\mathcal {J}$ in $\mathcal {A}$. The coercivity of $\mathcal {J}$ with respect to $\varvec{\nabla } \varvec{G}$ and the fact that $\{ \varvec{G}_j \}_{j \in \mathbb {N}}$ is bounded in $L^{\infty }$ implies that $\{ \varvec{G}_j \}_{j \in \mathbb {N}}$ is bounded in $H^1 (\Omega _0, \mathbb {R}^{N\times N})$. Thus, we can extract a subsequence (not relabelled) such that $\varvec{G}_j \rightharpoonup \varvec{G}$ in $H^1$ and $\varvec{G}_j \rightarrow \varvec{G}$ in $L^2$ and a.e., for some $\varvec{G} \in H^1$. Since *K* is closed, we see that $\varvec{G} (\varvec{X}) \in K$ for a.e. $\varvec{X} \in \Omega _0$. Thanks to (b), for a.e. $\varvec{X} \in \Omega _0$,

$$\begin{aligned} \phi (\varvec{X}, \varvec{G} (\varvec{X})) \le \liminf _{j \rightarrow \infty } \phi (\varvec{X}, \varvec{G}_j (\varvec{X})), \end{aligned}$$

and so, by Fatou’s lemma,

$$\begin{aligned} \int _{\Omega _0} \phi (\varvec{X}, \varvec{G} (\varvec{X})) \, d\varvec{X} \le \liminf _{j \rightarrow \infty } \int _{\Omega _0} \phi (\varvec{X}, \varvec{G}_j (\varvec{X})) \, d\varvec{X}. \end{aligned}$$

By the weak convergence in $H^1$,

$$\begin{aligned} \int _{\Omega _0} \Vert \varvec{\nabla } \varvec{G} (\varvec{X}) \Vert ^2 \, d\varvec{X} \le \liminf _{j \rightarrow \infty } \int _{\Omega _0} \Vert \varvec{\nabla } \varvec{G}_j (\varvec{X}) \Vert ^2 \, d\varvec{X}. \end{aligned}$$

In addition, we ought to check that $\varvec{G} \in \mathcal {A}$.

Let us check that $\varvec{G}$ is the gradient of an a.e. injective map. To this aim, we use that for each $j \in \mathbb {N}$ we have $\varvec{G}_j = \varvec{\nabla } \varvec{\Phi }_{\textrm{g}j}$ for some Sobolev map $\varvec{\Phi }_{\textrm{g}j}: \Omega _0 \rightarrow \mathbb {R}^N$ that is injective a.e. Without loss of generality, we can assume that

$$\begin{aligned} \int _{\Omega _0} \varvec{\Phi }_{\textrm{g}j} = 0. \end{aligned}$$

As $\varvec{G}_j \in H^1$, we have that $\varvec{\Phi }_{\textrm{g}j} \in H^2$. By the Poincaré–Wirtinger inequality,

$$\begin{aligned} \left\| \varvec{\Phi }_{\textrm{g}j} \right\| _{L^2} \le C \left\| \varvec{G}_j \right\| _{L^2}. \end{aligned}$$

Therefore, the sequence $\{\varvec{\Phi }_{\textrm{g}j} \}_{j \in \mathbb {N}}$ is bounded in $H^2$, so we can extract a subsequence weakly convergent in $H^2$ to some $\varvec{\Phi }_{\textrm{g}} \in H^2$. Moreover, we can assume that the convergence $\varvec{\Phi }_{\textrm{g}j} \rightarrow \varvec{\Phi }_{\textrm{g}}$ also holds a.e. As $\varvec{G}_j \rightharpoonup \varvec{G}$ in $H^1$, we have that $\varvec{G} = \varvec{\nabla } \varvec{\Phi }_{\textrm{g}}$. Let us see that $\varvec{\Phi }_{\textrm{g}}$ is injective. For this, we can apply Proposition 3.3, according to which it is enough to show that

$$\begin{aligned} \varvec{\Phi }_{\textrm{g}j} \in W^{1, N-1} , \quad {\text {cof}}\varvec{\nabla } \varvec{\Phi }_{\textrm{g}j} \in L^{\frac{N}{N-1}} , \quad \sup _{j \in \mathbb {N}} \left\| {\text {cof}}\varvec{\nabla } \varvec{\Phi }_{\textrm{g}j} \right\| _{L^1} < \infty , \\\varvec{\Phi }_{\textrm{g}j} \rightarrow \varvec{\Phi }_{\textrm{g}} \text { a.e.} , \quad \det \varvec{\nabla } \varvec{\Phi }_{\textrm{g}j} > 0 , \quad \det \varvec{\nabla } \varvec{\Phi }_{\textrm{g}j} \rightharpoonup {{\det \varvec{\nabla } \varvec{\Phi }_{\textrm{g}}}} \text { in } L^1 , \end{aligned}$$

with $\det \varvec{\nabla } \varvec{\Phi }_{\textrm{g}} > 0$ a.e. Those conditions are satisfied because of the convergence $\varvec{\Phi }_{\textrm{g}j} \rightharpoonup \varvec{\Phi }_{\textrm{g}}$ in $H^2$ and the Sobolev embeddings. Indeed, $\varvec{\Phi }_{\textrm{g}j} \in W^{1, N-1}$ because the embedding $H^2 \subset W^{1, N-1}$ is valid for $N \le 4$. In fact, $H^2 \subset W^{1, N}$ for $N \le 4$, so $\varvec{\nabla } \varvec{\Phi }_{\textrm{g}j} \in L^N$ and ${\text {cof}}\varvec{\nabla } \varvec{\Phi }_{\textrm{g}j} \in L^{\frac{N}{N-1}}$. Likewise, for some constants $c_i>0$,

$$\begin{aligned} \left\| {\text {cof}}\varvec{\nabla } \varvec{\Phi }_{\textrm{g}j} \right\| _{L^1} \le c_1 \left\| {\text {cof}}\varvec{\nabla } \varvec{\Phi }_{\textrm{g}j} \right\| _{L^{\frac{N}{N-1}}} \le c_2 \left\| \varvec{\nabla } \varvec{\Phi }_{\textrm{g}j} \right\| _{L^N}^{N-1} \le c_3 \left\| \varvec{\Phi }_{\textrm{g}j} \right\| _{H^2}^{N-1}, \end{aligned}$$

so $\sup _{j \in \mathbb {N}} \left\| {\text {cof}}\varvec{\nabla } \varvec{\Phi }_{\textrm{g}j} \right\| _{L^1} < \infty$. Convergence $\varvec{\Phi }_{\textrm{g}j} \rightarrow \varvec{\Phi }_{\textrm{g}} \text { a.e.}$ was shown earlier. Now, $\det \varvec{\nabla } \varvec{\Phi }_{\textrm{g}j} \ge m$ for some $m >0$, since $\varvec{G}_j \in K$ a.e. On the other hand, for $N \le 3$ the compact embedding $H^2 \subset W^{1, r}$ holds for $1 \le r < 6$, so $\varvec{\nabla } \varvec{\Phi }_{\textrm{g}j} \rightarrow \varvec{\nabla } \varvec{\Phi }_{\textrm{g}}$ in $L^r$ and, hence, $\det \varvec{\nabla } \varvec{\Phi }_{\textrm{g}j} \rightarrow \det \varvec{\nabla } \varvec{\Phi }_{\textrm{g}}$ in $L^s$ for all $s<2$. This implies the last condition since $\det \varvec{\nabla } \varvec{\Phi }_{\textrm{g}} \ge m$.

Another main step should focus on the first contribution to the cost given in terms of the Hausdorff distance $\rho _H \left( \Omega \left( \varvec{G}\right) ,\Omega _{\textrm{target}}\right)$, as well as the minimising relationship between $\varvec{\Phi }$ and $\varvec{G}$ in (2.10) and (2.8). To treat this step, it is mandatory to work with the minimiser $\varvec{\Phi }_j \in \mathbb {U}$ of (2.8) corresponding to $\varvec{G}_j$, for each $j \in \mathbb {N}$.

Take $\tilde{\varvec{\Phi }}\in \mathbb {U}$ such that $\Pi _{\varvec{G}} (\tilde{\varvec{\Phi }}) < \infty$. By minimality,

3.4 $$\begin{aligned} \int _{\Omega _0} W_{\varvec{G}_j} (\varvec{X}, \varvec{\nabla } \varvec{\Phi }_j) \, d\varvec{X} \le \int _{\Omega _0} W_{\varvec{G}_j} (\varvec{X}, \varvec{\nabla } \tilde{\varvec{\Phi }}) \, d\varvec{X} . \end{aligned}$$

By Lemma 3.5(c),

3.5 $$\begin{aligned} \int _{\Omega _0} W_{\varvec{G}_j} (\varvec{X}, \varvec{\nabla } \varvec{\Phi }_j) \, d\varvec{X} \ge C_1 \int _{\Omega _0} \left( \left\| \varvec{\nabla } \varvec{\Phi }_j \right\| ^p + \left\| {\text {cof}}\varvec{\nabla } \varvec{\Phi }_j \right\| ^q \right) d\varvec{X} - \frac{1}{C_1} \end{aligned}$$

for some $C_1>0$. On the other hand, using (3.3), we find that

3.6 $$\begin{aligned} W_{\varvec{G}_j} (\varvec{X}, \varvec{\nabla } \tilde{\varvec{\Phi }} (\varvec{X})) \le C_2 \left( 1 + W_0 (\varvec{\nabla } \tilde{\varvec{\Phi }} (\varvec{X})) \right) \end{aligned}$$

for some $C_2 > 0$. This inequality, together with (3.4) and (3.5) shows that $\{ \varvec{\nabla } \varvec{\Phi }_j \}_{j \in \mathbb {N}}$ is bounded in $L^p$ and $\{ {\text {cof}}\varvec{\nabla } \varvec{\Phi }_j \}_{j \in \mathbb {N}}$ is bounded in $L^q$.

As in the proof of Theorem 3.4, we obtain the existence of a $\varvec{\Phi } \in \mathbb {U}$ such that $\varvec{\Phi }_j \rightharpoonup \varvec{\Phi }$ in $W^{1,p}$, together with

$$\begin{aligned} \varvec{\Phi }_j \rightarrow \varvec{\Phi } \quad \text {a.e.,} \quad {\text {cof}}\varvec{\nabla } \varvec{\Phi }_j \rightharpoonup {\text {cof}}\varvec{\nabla } \varvec{\Phi } \quad \text {in } L^q(\Omega _0), \quad \det \varvec{\nabla } \varvec{\Phi }_j \rightharpoonup \det \varvec{\nabla } \varvec{\Phi } \quad \text {in } L^1 (\Omega _0). \end{aligned}$$

By Lemma 3.2,

3.7 $$\begin{aligned} \int _{\Omega _0} W_{\varvec{G}} (\varvec{X}, \varvec{\nabla } \varvec{\Phi }) \,d\varvec{X} \le \liminf _{j\rightarrow \infty } \int _{\Omega _0} W_{\varvec{G}_j} (\varvec{X}, \varvec{\nabla } \varvec{\Phi }_j) \,d\varvec{X} . \end{aligned}$$

On the other hand, using dominated convergence, bound (3.6) and Lemma 3.8, we find that

3.8 $$\begin{aligned} \lim _{j \rightarrow \infty } \int _{\Omega _0} W_{\varvec{G}_j} (\varvec{X}, \varvec{\nabla } \tilde{\varvec{\Phi }}) \, d\varvec{X} = \int _{\Omega _0} W_{\varvec{G}} (\varvec{X}, \varvec{\nabla } \tilde{\varvec{\Phi }}) \, d\varvec{X} . \end{aligned}$$

Putting together (3.4), (3.7) and (3.8) we conclude that

$$\begin{aligned} \int _{\Omega _0} W_{\varvec{G}} (\varvec{X}, \varvec{\nabla } \varvec{\Phi }) \, d\varvec{X} \le \int _{\Omega _0} W_{\varvec{G}} (\varvec{X}, \varvec{\nabla } \tilde{\varvec{\Phi }}) \, d\varvec{X}, \end{aligned}$$

and the arbitrariness of $\tilde{\varvec{\Phi }}$ in $\mathbb {U}$ implies that $\varvec{\Phi }$ is a minimiser of (2.8) in $\mathbb {U}$ for our limit $\varvec{G}$.

The final ingredient is provided by Proposition 3.7. Indeed, its assumptions have already been checked, so

$$\begin{aligned} \rho _H \left( \Omega \left( \varvec{G}\right) ,\Omega _{\textrm{target}} \right) \le \liminf _{j \rightarrow \infty } \rho _H \left( \Omega \left( \varvec{G}_j \right) ,\Omega _{\textrm{target}} \right) . \end{aligned}$$

Altogether, we see that

$$\begin{aligned} \mathcal {J} (\varvec{G}) \le \liminf _{j \rightarrow \infty } \mathcal {J} (\varvec{G}_j), \end{aligned}$$

and the proof is finished. $\square$

#### Remark 3.10

The same conclusion of Theorem 3.9 holds if we replace the term $\Vert \varvec{\nabla } \varvec{G} (\varvec{X}) \Vert$ with the term $\Vert \hat{\varvec{G}} (\varvec{X}) \Vert$; see (2.16) and note that $l>0$ is given. In this case the new functional is

$$\begin{aligned} \mathcal {J} (\varvec{G}) = \rho _H \left( \Omega \left( \varvec{G}\right) ,\Omega _{\textrm{target}}\right) + \int _{\Omega _0} \left( \alpha \Vert \hat{\varvec{G}} (\varvec{X}) \Vert ^2 + \phi (\varvec{X}, \varvec{G} (\varvec{X})) \right) d \varvec{X}. \end{aligned}$$

We explain the only steps of the proof that differ from that of Theorem 3.9. Let $\{ \varvec{G}_j \}_{j \in \mathbb {N}}$ be a minimising sequence of $\mathcal {J}$ in $\mathcal {A}$. Then $\{ \varvec{G}_j \}_{j \in \mathbb {N}}$ is bounded in $L^2 (\Omega )$, so there exists $\varvec{G} \in L^2 (\Omega _0)$ such that, for a subsequence, $\varvec{G}_j \rightharpoonup \varvec{G}$ in $L^2 (\Omega )$. Let $\hat{\varvec{G}}_j \in H^2 (\Omega _0)$ be the solution of (2.16) with right-hand side $\varvec{G}_j$, and $\hat{\varvec{G}} \in H^2 (\Omega _0)$ the solution with right-hand side $\varvec{G}$. By standard elliptic regularity theory (see, e.g., [26, Prop. 9.26]), $\hat{\varvec{G}}_j \rightharpoonup \hat{\varvec{G}}$ in $H^2 (\Omega _0)$, and, hence, $\hat{\varvec{G}}_j \rightarrow \hat{\varvec{G}}$ in $H^1 (\Omega _0)$. From here, the rest of the proof is identical to that of Theorem 3.9.

## The Theory of Morphoelasticity

Our main source in this section is the book [12]. The basic principle of morphoelasticity postulates a multiplicative decomposition of the deformation gradient in the form

4.1 $$\begin{aligned} \varvec{F} (\varvec{X}) = \varvec{A} (\varvec{X}) \varvec{G} (\varvec{X}). \end{aligned}$$

This decomposition replaces (2.3), the main difference being that there is no intermediate mappings $\varvec{\Phi }_{\text {g}}$ and $\varvec{\Phi }_{\text {e}}$ to account for growth and elastic deformation, respectively: tensors $\varvec{A}$ and $\varvec{G}$ are not associated with any deformation. “The growth tensor $\varvec{G}$ takes the initial configuration to a virtual stress-free state that may be incompatible. Then, a local elastic tensor $\varvec{A}$ restores compatibility of the body and enforces the boundary conditions and body forces so that the body is in a compatible configuration in mechanical equilibrium” ([12, p. 355]). Yet, the elastic constitutive law is formulated through an internal energy density $W=W(\varvec{A})$ that depends only on the elastic deformation tensor $\varvec{A}=\varvec{F} \varvec{G}^{-1}$, i.e., (2.4) and (2.5) are still valid, with $\varvec{F}=\varvec{\nabla }\varvec{\Phi }$. The rest of Sect. 2.1 is also valid word by word.

The idea of a decomposition of the form (4.1) in Mechanics can be traced back to the mid of the last century and first appeared in the contexts of anelasticity, placticity, dislocations, thermoelasticity and, more recently, biomechanics and growth mechanics. A survey of the history of this decomposition can be found in [27]. In fact, the recent papers [28, 29] explore when “virtual, incompatible” state actually exists as a global intermediate configuration.

Since our preceding analysis does not rely in any way on the fact that growth tensor $\varvec{G}$ comes from a gradient, i.e., is globally compatible, all of our previous results and discussions are correct in this new setting as well. In particular, the shape programming problem is formally the same as (2.10):

4.2 $$\begin{aligned} {\left\{ \begin{array}{ll} \text {Minimise in } \varvec{G}: &{} \mathcal {J} \left( \varvec{G} \right) := \rho _H \left( \Omega \left( \varvec{G}\right) ,\Omega _{\textrm{target}}\right) + \mathcal {C} \left( \varvec{G}\right) \\ \text {subject to:} &{} \varvec{G}\in H^1\left( \Omega _0; K \right) , \ \Omega \left( \varvec{G}\right) = \varvec{\Phi }\left( \Omega _0\right) ,\\ &{} \text {with } \varvec{\Phi }\in \mathbb {U}\text { a minimiser of } (2.8). \end{array}\right. } \end{aligned}$$

Notice that the only difference with the gradient case is the non-occurrence of the constraint that $\varvec{G}$ must be the gradient of an a.e. injective Sobolev map, so the proof of the existence result in this case would be shorter and less technical than that of Theorem 3.9. For record purposes, we state the main existence theorem in this setting.

### Theorem 4.1

Let $W_0: \mathbb {R}^{N\times N}_+ \rightarrow [0, \infty ]$ satisfy conditions (W1)–(W4) of Sect. 2.3. Let $K \subset \mathbb {R}^{N\times N}_+$ be compact. Let $\varvec{f} \in L^2 (\Omega _0, \mathbb {R}^N)$ and $\varvec{s}_0 \in L^2 (\Gamma _{0_N}, \mathbb {R}^N)$. Let $\phi : \Omega _0 \times \mathbb {R}^{N\times N}\rightarrow [0, \infty ]$ satisfy:

- $$\phi $$ is $$\mathcal {L} \times \mathcal {B}$$-measurable.
- $\phi (\varvec{X}, \cdot )$ is lower semicontinuous for a.e. $\varvec{X} \in \Omega _0$.

Assume that $\mathbb {U}\ne \varnothing$. Let $\alpha >0$. Define

$$\begin{aligned} \mathcal {J} (\varvec{G}) = \rho _H \left( \Omega \left( \varvec{G}\right) ,\Omega _{\textrm{target}}\right) + \int _{\Omega _0} \left( \alpha \Vert \varvec{\nabla } \varvec{G} (\varvec{X}) \Vert ^2 + \phi (\varvec{X}, \varvec{G} (\varvec{X})) \right) d \varvec{X} \end{aligned}$$

in

$$\begin{aligned} \mathcal {A} =&\{ \varvec{G}\in H^1\left( \Omega _0; K\right) : \Pi _{\varvec{G}} \text { is not identically infinity in } \mathbb {U}, \\&\Omega \left( \varvec{G}\right) = \varvec{\Phi }\left( \Omega _0\right) , \text {with } \varvec{\Phi } \text { a minimiser of } \Pi _{\varvec{G}} \text { in } \mathbb {U}\} . \end{aligned}$$

Assume that $\mathcal {A} \ne \varnothing$ and that $\mathcal {J}$ is not identically infinity in $\mathcal {A}$. Then there exists a minimiser of $\mathcal {J}$ in $\mathcal {A}$.

### Remark 4.2

According to (2.16) and Remarks 3.10, the same conclusion of Theorem holds if we replace the term $\Vert \varvec{\nabla } \varvec{G} (\varvec{X}) \Vert$ with the term $\Vert \hat{\varvec{G}} (\varvec{X}) \Vert$ for a given $l > 0$. In other words, the same result holds for the functional

$$\begin{aligned} \mathcal {J} (\varvec{G}) = \rho _H \left( \Omega \left( \varvec{G}\right) ,\Omega _{\textrm{target}}\right) + \int _{\Omega _0} \left( \alpha \Vert \hat{\varvec{G}} (\varvec{X}) \Vert ^2 + \phi (\varvec{X}, \varvec{G} (\varvec{X})) \right) d \varvec{X}. \end{aligned}$$

We anticipated in Sect. 2.4 that by isotropy one can work with $\varvec{C}_{\textrm{g}} = \varvec{G}^T \varvec{G}$, instead of $\varvec{G}$ as the main variable. In Sects. 2 and 3 we opted for $\varvec{G}$ so as not to deal with the constraint that $\varvec{C}_{\textrm{g}}$ is a metric tensor, but in the context of this section, the theory of morphoelasticity only requires that $\varvec{C}_{\textrm{g}}$ is a field of positive definite symmetric matrices. Let us see why isotropy allows working with $\varvec{C}_{\textrm{g}}$. Recall from Sect. 2.1 that the stored energy function of the material is $W_0$ and that, once the growth takes place, the total energy of the deformation is given by the integral (2.4), where the new stored-energy function is $W_{\varvec{G}}$ given by (2.5). Now, if a growth tensor $\varvec{G}_1$ changes to $\varvec{G}_2 = \varvec{R} \varvec{G}_1$ for some $\varvec{R} \in SO(N)$, then, using (2.5) and the isotropy of $W_0$, we find that

$$\begin{aligned} W_{\varvec{G}_2} (\varvec{X}, \varvec{F}) = W_0 (\varvec{F} \varvec{G}_1 (\varvec{X})^{-1} \varvec{R}^{-1}) \det \left( \varvec{R} \varvec{G}_1 (\varvec{X}) \right) = W_{\varvec{G}_1} (\varvec{X}, \varvec{F}). \end{aligned}$$

Thus, by polar decomposition, the dependence of $W_{\varvec{G}}$ on $\varvec{G}$ is only through $\varvec{C}_{\textrm{g}}$. Likewise, the cost functional $\mathcal {C}$ should be isotropic (as well as frame-indifferent). The required conditions for that were written in [13, Sect. 2(a)]. In the particular case of functionals of the form

$$\begin{aligned} \int _{\Omega _0} \left( \alpha \Vert \varvec{\nabla } \varvec{C}_{\textrm{g}} (\varvec{X}) \Vert ^2 + \phi (\varvec{X}, \varvec{C}_{\textrm{g}} (\varvec{X})) \right) d \varvec{X} \end{aligned}$$

(as in Theorem 4.3 below), the condition for $\phi$ is

4.3 $$\begin{aligned} \phi (\varvec{X}, \varvec{C}) = \phi (\varvec{X}, \varvec{R} \varvec{C} \varvec{R}^T) \end{aligned}$$

for a.e. $\varvec{X} \in \Omega _0$, all symmetric definite positive $\varvec{C} \in \mathbb {R}^{N\times N}$ and all $\varvec{R} \in SO(N)$.

Since this is the framework of the numerical experiments in the next section, we present the programming problem in the language of (4.2):

4.4 $$\begin{aligned} {\left\{ \begin{array}{ll} \text {Minimise in } \varvec{C}_{\textrm{g}}: &{} \mathcal {J} \left( \varvec{C}_{\textrm{g}} \right) := \rho _H \left( \Omega \left( \varvec{G}\right) ,\Omega _{\textrm{target}}\right) + \mathcal {C} \left( \varvec{C}_{\textrm{g}} \right) \\ \text {subject to:} &{} \varvec{C}_{\textrm{g}} \in H^1\left( \Omega _0; K \right) , \ \Omega \left( \varvec{G}\right) = \varvec{\Phi }\left( \Omega _0\right) \text {, with } \varvec{G} = \varvec{C}_{\textrm{g}}^{\frac{1}{2}} \\ &{} \text {and } \varvec{\Phi }\in \mathbb {U}\text { a minimiser of } (2.8). \end{array}\right. } \end{aligned}$$

In this case, $\mathcal {C} \left( \varvec{C}_{\textrm{g}} \right)$ can take the form (2.14), (2.15) or the general form given in the following theorem, which we present without proof.

### Theorem 4.3

Let $W_0: \mathbb {R}^{N\times N}_+ \rightarrow [0, \infty ]$ be isotropic and satisfy conditions (W1)–(W4) of Sect. 2.3. Let *K* be a compact subset of symmetric positive definite $N \times N$ matrices. Let $\varvec{f} \in L^2 (\Omega _0, \mathbb {R}^N)$ and $\varvec{s}_0 \in L^2 (\Gamma _{0_N}, \mathbb {R}^N)$. Let $\phi : \Omega _0 \times \mathbb {R}^{N\times N}\rightarrow [0, \infty ]$ satisfy (a)–(b) of Theorem 4.1 as well as (4.3). For each $\varvec{C}_{\textrm{g}}$, let $\varvec{G}$ be its symmetric positive definite square root. Assume that $\mathbb {U}\ne \varnothing$. Let $\alpha >0$. Define

$$\begin{aligned} \mathcal {J} (\varvec{C}_{\textrm{g}}) = \rho _H \left( \Omega \left( \varvec{G}\right) ,\Omega _{\textrm{target}}\right) + \int _{\Omega _0} \left( \alpha \Vert \varvec{\nabla } \varvec{C}_{\textrm{g}} (\varvec{X}) \Vert ^2 + \phi (\varvec{X}, \varvec{C}_{\textrm{g}} (\varvec{X})) \right) d \varvec{X} \end{aligned}$$

in

$$\begin{aligned} \mathcal {A} =&\{ \varvec{C}_{\textrm{g}} \in H^1\left( \Omega _0; K\right) : \Pi _{\varvec{G}} \text { is not identically infinity in } \mathbb {U}, \\&\Omega \left( \varvec{G}\right) = \varvec{\Phi }\left( \Omega _0\right) , \text {with } \varvec{\Phi } \text { a minimiser of } \Pi _{\varvec{G}} \text { in } \mathbb {U}\} . \end{aligned}$$

Assume that $\mathcal {A} \ne \varnothing$ and that $\mathcal {J}$ is not identically infinity in $\mathcal {A}$. Then there exists a minimiser of $\mathcal {J}$ in $\mathcal {A}$.

In the above theorem we have taken $\varvec{G}$ as the only symmetric positive definite square root of $\varvec{C}_{\textrm{g}}$, but, as explained earlier, any square root of $\varvec{C}_{\textrm{g}}$ gives rise to the same problem. Indeed, $\Pi _{\varvec{G}_1} = \Pi _{\varvec{G}_2}$ if $\varvec{G}_2 = \varvec{R} \varvec{G}_1$ for some $\varvec{R} \in SO(N)$.

## Numerical Simulation

This section presents the numerical simulations of the shape-programmig problem analyzed in the previous sections, all done in dimension $N=3$. As noticed in [13] and explained in Sect. 4, under the presence of isotropy it is more convenient to implement numerically the growth-driven actuation by means of the right Cauchy–Green strain tensor $\varvec{C}_{\textrm{g}}=\varvec{G}^T\varvec{G}$. Indeed, this choice of the control variable introduces less nonlinearity into the formulation of the problem, which facilitates its numerical approximation. Moreover, in this section we use the theory of morphoelasticity, so it is not relevant in practise whether or not the growth tensor is a deformation gradient. All in all, this section addresses the numerical resolution of the shape-programming problem (4.4).

The layout of this section is as follows. In Sect. 5.1, after parametrising $\varvec{C}_{\textrm{g}}$ in terms of its eigenvalues and eigenvectors, we find an equivalent formulation of (4.2), which is more amenable to computing the gradients that are required in gradient-based optimisation algorithms. We also present the numerical scheme. In Sect. 5.2 we perform several numerical experiments. A set of experiments deals with an initial geometry resembling a beam and another set resembling a shell. For these experiments it is enough to control the eigenvalues of the tensor $\varvec{C}_{\textrm{g}}$, while keeping the eigenvectors fixed. In the final example we show that, when the initial configuration is a cube and the final configuration is a cylinder, it is necessary to consider both eigenvalues and eigenvectors as design variables to achieve a satisfactory match between the final and the target configurations.

To accomodate the notation to that widely used in Computational Mechanics, from now on in this section, we denote by $\varvec{H} = {\text {cof}}\varvec{F}$ and by $J = \det \varvec{F}$.

### Numerical Resolution Method

#### Parametrisation of the Growth Tensor

Consider the following version of the Mooney–Rivlin density energy presented in (2.13):

$$\begin{aligned} W_0(\varvec{F})=\frac{\mu _1}{2}\vert \vert \varvec{F}\vert \vert ^2 + \frac{\mu _2}{2}\vert \vert \varvec{H}\vert \vert ^2 - (\mu _1+2\mu _2)\log J + \frac{\lambda }{2}(J-1)^2. \end{aligned}$$

This energy is isotropic, so it is valid to work with $\varvec{C}_g$ instead of $\varvec{G}$. The actuated energy density $\psi (\varvec{F},\varvec{C}_g)$, equivalent to $W_{\varvec{G}}$ in (2.5), adopts the expression

5.1 $$\begin{aligned} \begin{aligned} \psi (\varvec{F},\varvec{C}_{\textrm{g}})&=\frac{\mu _1}{2}\left( \det \varvec{C}_{\textrm{g}}\right) ^{-1/2}{{\,\textrm{tr}\,}}\left( \varvec{F}^T\varvec{F}{\text {cof}}\varvec{C}_{\textrm{g}}\right) +\frac{\mu _2}{2}\left( \det \varvec{C}_{\textrm{g}}\right) ^{-1/2}{{\,\textrm{tr}\,}}\left( \varvec{H}^T\varvec{H}\varvec{C}_{\textrm{g}}\right) \\&\quad -\left( \mu _1+2\mu _2\right) \left( \det \varvec{C}_{\textrm{g}}\right) ^{1/2}\log (J \left( \det \varvec{C}_{\textrm{g}}\right) ^{-1/2})\\&\quad + \frac{\lambda }{2}\left( \det \varvec{C}_{\textrm{g}}\right) ^{1/2}\Big (J \left( \det \varvec{C}_{\textrm{g}}\right) ^{-1/2} - 1\Big )^2. \end{aligned} \end{aligned}$$

The eigenvalue decomposition of $\varvec{C}_{\textrm{g}}$ is given by

$$\begin{aligned} \varvec{C}_{\textrm{g}}=\varvec{V}\varvec{\Lambda }\varvec{V}^T , \qquad \varvec{\Lambda }=\begin{bmatrix} \lambda _1 &{}\quad 0 &{}\quad 0\\ 0 &{}\quad \lambda _2 &{}\quad 0\\ 0 &{}\quad 0 &{}\quad \lambda _3 \end{bmatrix} , \end{aligned}$$

where the ortonormal eigenvectors are encapsulated in the columns of $\varvec{V}$, i.e., $$\varvec{V}=\begin{bmatrix} \varvec{v}_1&\varvec{v}_2&\varvec{v}_3 \end{bmatrix}$$, whilst $\varvec{\Lambda }$ encodes the eigenvalues $\{\lambda _1,\lambda _2,\lambda _3\}$ of $\varvec{C}_{\textrm{g}}$. Thus, $\varvec{C}_{\textrm{g}}$ is rewritten as

5.2 $$\begin{aligned} \varvec{C}_{\textrm{g}} = \sum _{i=1}^3\lambda _i \varvec{v}_i\otimes \varvec{v}_i. \end{aligned}$$

Positive definiteness of $\varvec{C}_{\textrm{g}}$ entails positivity of the eigenvalues $\lambda _1,\lambda _2,\lambda _3$. Moreover, in some applications one may wish to impose incompressibility in $\varvec{C}_{\textrm{g}}$, which is modelled by condition $\det \varvec{C}_{\textrm{g}}=1$ and is equivalent to a restriction only on $\varvec{\Lambda }$, namely, $\det \varvec{\Lambda }=1$. This can be accomplished, for instance, by parametrising $\varvec{\Lambda }$ as

$$\begin{aligned} \varvec{\Lambda } = \begin{bmatrix} \lambda _1 &{}\quad 0 &{}\quad 0\\ 0 &{}\quad \lambda _2 &{}\quad 0\\ 0 &{}\quad 0 &{}\quad \frac{1}{\lambda _1 \lambda _2} \end{bmatrix} , \end{aligned}$$

although we have not included any experiment in this context.

It remains to define the eigenvectors $\{\varvec{v}_1,\varvec{v}_2,\varvec{v}_3\}$. A possibility for that is to define the matrix $\varvec{V}$ by using the Rodrigues formula, according to which $\varvec{V}$ is parametrised in terms of a unitary vector $\varvec{k}\in \mathbb {R}^3$, and a rotation angle $\theta _3\in [0,2\pi [$ around $\varvec{k}$ as

5.3 $$\begin{aligned} \varvec{V} = \varvec{V}(\varvec{k},\theta _3)= \varvec{I} - \sin \theta _3\varvec{K} + \left( 1 -\cos \theta _3\right) \varvec{K}\varvec{K};\qquad \varvec{K}=\varvec{\mathcal {E}}:\varvec{k}, \end{aligned}$$

where $\varvec{\mathcal {E}}$ is the third-order alternating tensor (or Levi–Civita tensor), and $\varvec{k}$ is defined through a spherical parametrisation as

5.4 $$\begin{aligned} \varvec{k} = \begin{bmatrix} \cos \theta _1 \sin {\theta _2} {{,}} \ \sin \theta _1 \sin {\theta _2} {{,}} \ \cos \theta _2 \end{bmatrix}^T;\qquad \theta _1\in [0,2\pi [;\qquad \theta _2\in [0,\pi [ . \end{aligned}$$

introducing the above parametrisation (5.4) into (5.3) yields

5.5 $$\begin{aligned} \begin{aligned} \varvec{v}_1&= \begin{bmatrix} (\cos \theta _3 - 1)\left( \cos ^2\theta _2 + \sin ^2\theta _2 \sin ^2\theta _1\right) + 1 \\ \cos \theta _2\sin \theta _3 - \cos \theta _1\sin ^2\theta _2\sin \theta _1(\cos \theta _3 - 1) \\ \sin \theta _2\left( \sin \theta _3\sin \theta _1 - \cos \theta _2\cos \theta _1(\cos \theta _3 - 1)\right) \end{bmatrix} , \\ \varvec{v}_2&= \begin{bmatrix} -\cos \theta _2\sin \theta _3 - \cos \theta _1\sin ^2\theta _2\sin \theta _1(\cos \theta _3 - 1)\\ (\cos \theta _3 - 1)\left( \cos ^2\theta _2 + \cos ^2\theta _1\sin ^2\theta _2\right) + 1\\ \sin \theta _2\left( \sin \theta _3\cos \theta _1 - \cos \theta _2\sin \theta _1(\cos \theta _3 - 1)\right) \\ \end{bmatrix} , \\ \varvec{v}_3&= \begin{bmatrix} \sin \theta _2\left( \sin \theta _3\sin \theta _1 - \cos \theta _2\cos \theta _1(\cos \theta _3 - 1)\right) \\ -\sin \theta _2\left( \sin \theta _3\cos \theta _1 + \cos \theta _2\sin \theta _1(\cos \theta _3 - 1)\right) \\ (\cos \theta _3 - 1)\left( \sin ^2\theta _2\sin ^2\theta _1 + \cos ^2\theta _1\sin ^2\theta _2\right) + 1 \end{bmatrix} . \end{aligned} \end{aligned}$$

#### An Equivalent Formulation of the Optimisation Problem

This new set of design variables, $\varvec{\lambda }=\{\lambda _1,\lambda _2,\lambda _3\}$ and $\varvec{\theta }=\{\theta _1,\theta _2,\theta _3\}$, allows us to consider the new optimisation problem5.6where, in analogy to (2.16), the fields

$$\begin{aligned} \hat{\varvec{\lambda }}(\varvec{\lambda })=\{\hat{\lambda }_1(\lambda _1),\hat{\lambda }_2(\lambda _2),\hat{\lambda }_3(\lambda _3)\} \quad \text {and} \quad \hat{\varvec{\theta }}(\varvec{\theta })=\{\hat{\theta }_1(\theta _1),\hat{\theta }_2(\theta _2),\hat{\theta }_3(\theta _3)\} \end{aligned}$$

are regularised versions of $\varvec{\lambda }$ and $\varvec{\theta }$, respectively. More precisely, for $1\le i\le 3$, the functions $\hat{\lambda }_i (\varvec{X})$ and $\hat{\theta }_i (\varvec{X})$ are in $H^1(\Omega _0)$

and are the solutions of the boundary value problems

5.7 $$\begin{aligned} \left\{ \begin{aligned} \hat{\lambda }_i - l^2 \Delta \hat{\lambda }_i&=\lambda _i,&\quad&\text {in}\,\, \Omega _0,\\ \nabla \hat{\lambda }_i\cdot \varvec{N}&=0,&\quad&\text {on} \,\,\partial \Omega _0, \end{aligned}\right. \qquad \left\{ \begin{aligned} \hat{\theta }_i - l^2 \Delta \hat{\theta }_i&=\theta _i,&\quad&\text {in}\,\, \Omega _0,\\ \nabla \hat{\theta }_i\cdot \varvec{N}&=0,&\quad&\text {on} \,\,\partial \Omega _0, \end{aligned}\right. \end{aligned}$$

where *l* is a length-scale parameter and $\varvec{N}$ is the normal to $\Omega _0$.

Note that in (5.6) we have not included the compexity functional $\mathcal {C}$ described in Sect. 2.4, although it can easily be incorporated. In any case, problem (5.6) fits in the theory of Theorem 4.3, just by putting $\phi = 0$, while the regularisation term based on the $L^2$ norm of $\varvec{\nabla } \varvec{C}_{\textrm{g}}$ is replaced by the regularisations $\hat{\varvec{\lambda }}$ and $\hat{\varvec{\theta }}$.

In addition, to account for the complexity of the actuation, we impose lower and upper pointwise bounds on $\lambda _i$, namely $\lambda _i^{lb}\le \lambda _i\le \lambda _i^{ub}$, $1\le i\le 3$, rather than an $L^2$-norm constraint. Indeed, those pointwise contraints are more effectively handled by constrained optimisation methods as they prevent from tuning the weighting parameters that appear in the complexity functional. Similarly, the angles $\theta _i$ are confined in suitable predefined intervals. Due to the maximum principle, the regularised variables $\hat{\lambda }_i$ and $\hat{\theta }_i$ satisfy the same bounds.

We present the existence result for (5.6).

##### Proposition 5.1

Let $F_{\varvec{\lambda }} \subset (0, \infty )^3$ and $F_{\varvec{\theta }} \subset \mathbb {R}^3$ be non-empty compact and convex sets. Then there exists a solution for Problem (5.6) under the bounds $\varvec{\lambda } \in F_{\varvec{\lambda }}$ and $\varvec{\theta } \in F_{\varvec{\theta }}$.

##### Proof

Let $\{ \left( \varvec{\lambda }_j, \varvec{\theta }_j \right) \}_{j \in \mathbb {N}}$ be a minimizing sequence. As $F_{\varvec{\lambda }}$ and $F_{\varvec{\theta }}$ are compact, the sequence $\{ \left( \varvec{\lambda }_j, \varvec{\theta }_j \right) \}_{j \in \mathbb {N}}$ is bounded in $L^{\infty } (\Omega _0)$ and, hence, in $L^q (\Omega _0)$ for all $q < \infty$. Therefore, there exists $\left( \varvec{\lambda }, \varvec{\theta } \right) \in L^q (\Omega _0)$ such that, for a subsequence, $\left( \varvec{\lambda }_j, \varvec{\theta }_j \right) \rightharpoonup \left( \varvec{\lambda }, \varvec{\theta } \right)$ in $L^q (\Omega _0)$. As $F_{\varvec{\lambda }}$ and $F_{\varvec{\theta }}$ are compact and convex, we get that $\varvec{\lambda } \in F_{\varvec{\lambda }}$ and $\varvec{\theta } \in F_{\varvec{\theta }}$. Let $\left( \hat{\varvec{\lambda }}_j, \hat{\varvec{\theta }}_j \right)$ be the solution of the corresponding problems (5.7) for right-hand side $\left( \varvec{\lambda }_j, \varvec{\theta }_j \right)$, and analogously for $\left( \hat{\varvec{\lambda }}, \hat{\varvec{\theta }} \right)$. By elliptic regularity theory, $\left( \hat{\varvec{\lambda }}_j, \hat{\varvec{\theta }}_j \right) \rightharpoonup \left( \hat{\varvec{\lambda }}, \hat{\varvec{\theta }} \right)$ in $W^{2,q} (\Omega _0)$ and, hence, $\left( \hat{\varvec{\lambda }}_j, \hat{\varvec{\theta }}_j \right) \rightarrow \left( \hat{\varvec{\lambda }}, \hat{\varvec{\theta }} \right)$ in $W^{1,q} (\Omega _0)$. For $i=1, 2, 3$, let $\hat{\varvec{v}}_i^j$ be the vectors (5.5) corresponding to the angles $\hat{\varvec{\theta }}_j$, and analogously for $\hat{\varvec{v}}_i$. Choosing $q > 3$, we can apply the result [30, Th. 3.1] on composition operators to conclude that $\hat{\varvec{v}}_i^j \rightarrow \hat{\varvec{v}}_i$ in $W^{1,q} (\Omega _0)$. The same result also implies $\hat{\varvec{v}}_i^j \otimes \hat{\varvec{v}}_i^j \rightarrow \hat{\varvec{v}}_i \otimes \hat{\varvec{v}}_i$ in $W^{1,q} (\Omega _0)$ and $\hat{\lambda }_i^j \hat{\varvec{v}}_i^j \otimes \hat{\varvec{v}}_i^j \rightarrow \hat{\lambda }_i \hat{\varvec{v}}_i \otimes \hat{\varvec{v}}_i$ in $W^{1,q} (\Omega _0)$, where $\hat{\lambda }_i^j$ are the components of $\hat{\varvec{\lambda }}_j$ and analogously for $\hat{\lambda }_i$. By (5.2), $\varvec{C}_{\textrm{g}}\left( \hat{\varvec{\lambda }}_j,\hat{\varvec{\theta }}_j \right) \rightarrow \varvec{C}_{\textrm{g}}\left( \hat{\varvec{\lambda }},\hat{\varvec{\theta }} \right)$ in $W^{1,q} (\Omega _0)$. The rest of the proof is identical to that of Theorems 3.9, 4.1 and 4.3. $\square$

#### Computation of Continuous Gradients

As is customary in gradient-based optimisation, in order to compute a descent direction, we use the standard Lagrangian method [31]. To this end, let us consider the Lagrangian $\mathscr {L}$ defined as

5.8 $$\begin{aligned} \mathscr {L}(\overline{\varvec{\Phi }},\overline{\varvec{p}},\overline{\varvec{\lambda }},\overline{\varvec{\theta }})=\mathcal {J}(\overline{\varvec{\Phi }}) - \int _{\Omega _0}\varvec{P}\left( \varvec{\nabla }\overline{\varvec{\Phi }},\overline{\varvec{\lambda }},\overline{\varvec{\theta }}\right) :\varvec{\nabla }\overline{\varvec{p}}\,d\varvec{X}, \end{aligned}$$

which is defined for $(\overline{\varvec{\Phi }},\overline{\varvec{p}},\overline{\varvec{\lambda }},\overline{\varvec{\theta }})\in H^1(\Omega _0;\mathbb {R}^3)\times H_0^1(\Omega _0;\mathbb {R}^3)\times H^1(\Omega _0; \mathbb {R}^3)\times H^1(\Omega _0; \mathbb {R}^3)$ and $\overline{\varvec{\Phi }}$ satisfying the boundary condition in $\Gamma _{0_D}$. For the strain energy in (5.1), the first Piola–Kirchhoff stress tensor $\varvec{P}=\partial _{\varvec{F}}\psi$ is

5.9 $$\begin{aligned} \begin{aligned} \varvec{P}(\varvec{F},\varvec{C}_{\textrm{g}}(\varvec{\lambda },\varvec{\theta }))&=\mu _1\left( \det \varvec{C}_{\textrm{g}}\right) ^{-1/2}\varvec{F}{\text {cof}}\varvec{C}_{\textrm{g}} + \mu _2\left( \det \varvec{C}_{\textrm{g}}\right) ^{-1/2}\Big (\varvec{H}\varvec{C}_{\textrm{g}}\times \varvec{F}\Big )\\&\quad +\Bigg (-\frac{\left( \mu _1+2\mu _2\right) \left( \det \varvec{C}_{\textrm{g}}\right) ^{1/2}}{J} + {\lambda }\Big (J \left( \det \varvec{C}_{\textrm{g}}\right) ^{-1/2} - 1\Big )\Bigg )\varvec{H} , \end{aligned} \end{aligned}$$

where $(\varvec{A}\times \varvec{B})_{iI}=\mathcal {E}_{ijk}\mathcal {E}_{IJK}A_{jJ}B_{kK}$, for $\varvec{A}$, $\varvec{B}\in \mathbb {R}^{3\times 3}$, and $\mathcal {E}_{IJK}$ represents the components of the third-order alternating tensor. Notice that $(\overline{\varvec{\Phi }},\overline{\varvec{p}},\overline{\varvec{\lambda }},\overline{\varvec{\theta }})$ are considered as independent variables in (5.8). The stationary condition of $\mathscr {L}$ (5.8) with respect to $\overline{\varvec{p}}$

5.10 $$\begin{aligned} {\partial _{\overline{\varvec{p}}}}{\mathcal {L}}(\overline{\varvec{\Phi }},\overline{\varvec{p}},\overline{\varvec{\lambda }},\overline{\varvec{\theta }})(\varvec{v}) =\varvec{0} , \quad \text {for all }\varvec{v}\in H^1\left( \Omega _0, \mathbb {R}^3\right) \text { with } \varvec{v} = \varvec{0} \text { on } \Gamma _{0_D}, \end{aligned}$$

yields indeed the stationary point of the functional $\Pi _{\varvec{G}}$ in (2.8), namely the weak form of the traslational equilibrium, since

5.11 $$\begin{aligned} {\partial _{\overline{\varvec{p}}}}{\mathcal {L}}(\overline{\varvec{\Phi }},\overline{\varvec{p}},\overline{\varvec{\lambda }},\overline{\varvec{\theta }})(\varvec{v})=- \partial _{\overline{\varvec{\Phi }}}{\Pi }(\overline{\varvec{\Phi }},\overline{\varvec{p}},\overline{\varvec{\lambda }},\overline{\varvec{\theta }})(\varvec{v}) =\int _{\Omega _0}\varvec{P}(\varvec{\nabla }\overline{\varvec{\Phi }},\overline{\varvec{\lambda }},\overline{\varvec{\theta }}):\varvec{\nabla }\varvec{v}\,d\varvec{X} . \end{aligned}$$

Equation (5.10) with expression (5.11) is nonlinear. A consistent linearisation of (5.11) has been carried out by means of the standard Newton–Raphson method in order to obtain the deformed configuration $\varvec{x}=\varvec{\Phi }(\varvec{X})$. Similarly, the stationary condition of the Lagrangian $\mathscr {L}$ with respect to $\overline{\varvec{\Phi }}$ yields

5.12 $$\begin{aligned} \begin{aligned} {\partial _{\overline{\varvec{\Phi }}}}{\mathcal {L}}(\overline{\varvec{\Phi }},\overline{\varvec{p}},\overline{\varvec{\lambda }},\overline{\varvec{\theta }})(\varvec{v})&=\partial _{\overline{\varvec{\Phi }}}{\mathcal {J}}(\overline{\varvec{\Phi }})(\varvec{v}) - \int _{\Omega _0}\varvec{\nabla }\varvec{v}:\varvec{\mathcal {C}}(\varvec{\nabla }\overline{\varvec{\Phi }},\overline{\varvec{\lambda }},\overline{\varvec{\theta }}):\varvec{\nabla }\overline{\varvec{p}}\,d\varvec{X}=\varvec{0}, \end{aligned} \end{aligned}$$

where $\varvec{\mathcal {C}}$ represents the fourth order elasticity tensor, defined as

$$\begin{aligned} \varvec{\mathcal {C}}(\varvec{F})=\varvec{\nabla }^2_{\varvec{FF}}\psi , \end{aligned}$$

which takes the form

$$\begin{aligned} \begin{aligned} \varvec{\nabla }_{\varvec{FF}}^2\psi&=\partial ^2_{\varvec{FF}}W + \varvec{F}\times \partial ^2_{\varvec{HH}}W \times \varvec{F} + \partial ^2_{{JJ}}W \varvec{H}\otimes \varvec{H} + \varvec{\mathcal {I}}\times \left( \partial _{\varvec{H}}W + \partial _{{J}}W \varvec{F}\right) \end{aligned} \end{aligned}$$

being

$$\begin{aligned} \begin{aligned} \left( \partial ^2_{\varvec{FF}}W\right) _{iIjJ}&=\mu _1\delta _{ij}\left( {\text {cof}}\varvec{C}_{\textrm{g}}\right) _{IJ},\\ \left( \partial ^2_{\varvec{HH}}W\right) _{iIjJ}&=\mu _2\delta _{ij}\left( \varvec{C}_{\textrm{g}}\right) _{IJ},\\ \partial ^2_{JJ}W&= \frac{\left( \mu _1+2\mu _2\right) \left( \det \varvec{C}_{\textrm{g}}\right) ^{1/2}}{J^2} + {\lambda } \left( \det \varvec{C}_{\textrm{g}}\right) ^{-1/2} ,\\ \partial _{J}W&= -\frac{\left( \mu _1+2\mu _2\right) \left( \det \varvec{C}_{\textrm{g}}\right) ^{1/2}}{J} + {\lambda }\Big (J \left( \det \varvec{C}_{\textrm{g}}\right) ^{-1/2} - 1\Big ) ,\\ \partial _{\varvec{H}}W&=\mu _2\left( \det \varvec{C}_{\textrm{g}}\right) ^{-1/2}\varvec{H}\varvec{C}_{\textrm{g}}, \end{aligned} \end{aligned}$$

and

$$\begin{aligned} \begin{aligned} \left( \varvec{\mathcal {A}}\times \varvec{A}\right) _{iIjJ}&=\mathcal {A}_{iIpP}A_{qQ}\mathcal {E}_{jpq}\mathcal {E}_{JPQ},&\qquad \left( \varvec{A}\times \varvec{\mathcal {A}}\right) _{iIjJ}&=\mathcal {E}_{ipq}\mathcal {E}_{IPQ}A_{pP}\mathcal {A}_{qQjJ}, \\ \left( \varvec{A}\otimes \varvec{A}\right) _{iIjJ}&=A_{iI}A_{jJ} ,&\qquad \varvec{\mathcal {I}}_{iIjJ}&=\delta _{iI}\delta _{jJ}, \end{aligned} \end{aligned}$$

for $\varvec{A}\in \mathbb {R}^{3\times 3}$ and $\varvec{\mathcal {A}}\in \mathbb {R}^{3\times 3\times 3\times 3}$. From the linear equation in (5.12) it is therefore possible to obtain the adjoint state $\varvec{p}$.

The directional derivative of the Lagrangian $\mathscr {L}$ with respect to the design variables $\{\lambda _1,\lambda _2,\lambda _3\}$ and $\{\theta _1,\theta _2,\theta _3\}$ yields

$$\begin{aligned} \begin{aligned} {\partial _{\overline{{\lambda }}_i}}{\mathcal {L}}(\overline{{\varvec{\Phi }}},\overline{\varvec{p}},\overline{\varvec{\lambda }},\overline{\varvec{\theta }})( \delta {\lambda _i} )&= -\int _{\Omega _0}\partial _{\overline{\lambda }_i}\varvec{P}(\varvec{\nabla }\overline{\varvec{\Phi }},\overline{\varvec{\lambda }},\overline{\varvec{\theta }})( \delta {\lambda _i} ):\varvec{\nabla }\overline{\varvec{p}}\,d\varvec{X} , \\ {\partial _{\overline{{\theta }}_i}}{\mathcal {L}}(\overline{{\varvec{\Phi }}},\overline{\varvec{p}},\overline{\varvec{\lambda }},\overline{\varvec{\theta }})( \delta {\theta _i} )&= -\int _{\Omega _0}\partial _{\overline{\theta }_i}\varvec{P}(\varvec{\nabla }\overline{\varvec{\Phi }},\overline{\varvec{\lambda }},\overline{\varvec{\theta }})( \delta {\theta _i} ):\varvec{\nabla }\overline{\varvec{p}}\,d\varvec{X} , \end{aligned} \end{aligned}$$

which, for the specific expression for $\varvec{P}$ in (5.9), takes the following form

$$\begin{aligned} \begin{aligned} \partial _{\overline{\lambda }_i}\varvec{P}(\varvec{\nabla }\overline{\varvec{\Phi }},\overline{\varvec{\lambda }},\overline{\varvec{\theta }})( \delta {\lambda _i} ):\varvec{\nabla }\overline{\varvec{p}}&=:\varvec{v}_i\cdot \varvec{T}\varvec{v}_i , \\ \partial _{\overline{\theta }_i}\varvec{P}(\varvec{\nabla }\overline{\varvec{\Phi }},\overline{\varvec{\lambda }},\overline{\varvec{\theta }})( \delta {\theta _i} ):\varvec{\nabla }\overline{\varvec{p}}&=:\sum _{j=1}^3\partial _{\theta _i}\varvec{v}_j\cdot \left( \varvec{T} + \varvec{T}^T\right) \varvec{v}_j , \end{aligned} \end{aligned}$$

where the second order tensor $\varvec{T}$ is defined as

5.13 $$\begin{aligned} \begin{aligned} \varvec{T}&= -\frac{\mu _1}{2}(\det \varvec{C}_{\textrm{g}})^{-3/2}{{\,\textrm{tr}\,}}\left( \left( \varvec{F}{\text {cof}}\varvec{C}_{\textrm{g}}\right) ^T\varvec{\nabla }\varvec{p}\right) {\text {cof}}\varvec{C}_{\textrm{g}} \\&\quad +\mu _1(\det \varvec{C}_{\textrm{g}})^{-1/2}\Big (\varvec{C}_{\textrm{g}}\times \varvec{F}^T\varvec{\nabla }\varvec{p}\Big )\\&\quad -\frac{\mu _2}{2}(\det \varvec{C}_{\textrm{g}})^{-3/2}{{\,\textrm{tr}\,}}\left( \left( \varvec{H}\varvec{C}_{\textrm{g}}\right) ^T\left( \varvec{F}\times \varvec{\nabla }\varvec{p}\right) \right) {\text {cof}}\varvec{C}_{\textrm{g}}\\&\quad +\mu _2(\det \varvec{C}_{\textrm{g}})^{-1/2}\varvec{H}^T\Big (\varvec{F}\times \varvec{\nabla }\varvec{p}\Big )\\&\quad -{{\,\textrm{tr}\,}}\Big (\varvec{H}^T\varvec{\nabla }\varvec{p}\Big )\Big (\frac{\mu _1+2\mu _2}{2J (\det \varvec{C}_{\textrm{g}})^{1/2}}+\frac{\lambda J}{2(\det \varvec{C}_{\textrm{g}})^{3/2}}\Big ){\text {cof}}\varvec{C}_{\textrm{g}} . \end{aligned} \end{aligned}$$

#### Numerical Scheme

In order to clarify how the different equations featured in the current section have been embedded into a gradient algorithm, we summarise the steps involved in the optimisation.

Starting from an initial guess $(\varvec{\lambda }^0, \varvec{\theta }^0),$ we proceed with the loop:

(i) Solve the state equation (5.10) with expression (5.11), which yields the new deformation mapping $\varvec{\Phi }$ and the new deformed configuration $\Omega$.
(ii) Based on the new deformation mapping $\varvec{\Phi }$, compute the adjoint state field $\varvec{p}$ by means of (5.12).
(iii) Compute the objective function $\mathcal {J}(\varvec{\Phi }(\varvec{\lambda },\varvec{\theta }))$.
(iv) Compute descent directions for each of the design variables, namely $\partial _{\overline{\lambda }_i} \mathcal {L}$ and $\partial _{\overline{\theta }_i} \mathcal {L}$, $1\le i\le 3$.
(v) Pass $\mathcal {J}(\varvec{\Phi }(\varvec{\lambda },\varvec{\theta }))$, $\partial _{\overline{\lambda }_i} \mathcal {L}$ and $\partial _{\overline{\theta }_i} \mathcal {L}$, $1\le i\le 3$, to the gradient algorithm in order to determine the step size and hence, the new value of the design variables $(\lambda _1,\lambda _2,\lambda _3)$ and $(\theta _1,\theta _2,\theta _3)$.

##### Remark 5.2

Although the lower bound conditions $\lambda _i>0$ ensuring the positive definiteness of the tensor $\varvec{C}_{\textrm{g}}$ have not been explicitly included in the Lagrangian $\mathscr {L}$ in (5.8), any standard gradient-based algorithm such as the interior-point, can easily handle this type of constraint, by augmenting the Lagrangian $\mathscr {L}$ in (5.8) by means of the method of Lagrange multipliers. Evaluation of this additional term and of its derivatives with respect to the design variables $(\lambda _1,\lambda _2,\lambda _3)$ is therefore omitted in the derivations included in this section. In fact, as mentioned in Sect. 5.1.2, pointwise bounds on $\lambda _i$ and $\theta _i$, $1 \le i \le 3$, have been included in the optimisation method.

### Numerical Experiments

The objective of this section is to demonstrate the applicability of the proposed formulation in the context of shape morphing, i.e., determining the value of the optimal design variables $\{\lambda _1,\lambda _2,\lambda _3\}$ and $\{\theta _1,\theta _2,\theta _3\}$ following a gradient-based approach with the aim of attaining the closest growth-driven configuration to a given target configuration. As indicated in the introduction, one of the objectives of this paper is to present an alternative formulation to other analytical approaches that make use of simplifying assumptions such as the absence of boundary conditions, which permit to obtain a closed-form solution of the optimal growth tensor [32, 33]. We do not intend to claim that our formulation is more advantageous than others. On the contrary, as in other areas of continuum mechanics, analytical solutions can be extremely useful. Our purpose is to illustrate the possibility to apply inverse techniques for the optimal solution of this problem, which can address more generic situations than those covered by analytical approaches.

With regard to the constitutive model used, we consider the strain energy given by (5.1). In all the examples, the values of $\{\mu _1,\mu _2,\lambda \}$ are

$$\begin{aligned} \mu _1=0.5,\qquad \mu _2=0.5, \qquad \lambda = 3. \end{aligned}$$

In the first two examples (Sects. 5.2.1 and 5.2.2), we will advocate for a widely accepted formulation in engineering, according to which the eigenvectors $\{\varvec{v}_1,\varvec{v}_2,\varvec{v}_3\}$ of $\varvec{C}_{\textrm{g}}$ (see equation (5.2)) remain fixed, while only the eigenvalues $\{\lambda _1,\lambda _2,\lambda _3\}$ serve as the unknown fields to be determined analytically [32, 33]. Although this approach is less flexible compared to the more comprehensive formulation discussed in Sect. 5.1, it has exhibited reasonably positive outcomes in terms of achieving the target configuration. Typically, the eigenvectors of $\varvec{C}_{\textrm{g}}$ are considered coincident with the tangent vectors associated with the curvilinear coordinate system that describes the geometry of the initial solid configuration.

However, in the final example of Sect. 5.2.3, we will illustrate a scenario where incorporating additionally the eigenvectors as design variables (specifically, the three angular fields $\{\theta _1,\theta _2,\theta _3\}$ in equation (5.5)) allows for a higher degree of flexibility. This enhanced formulation enables a significantly better approximation to the target configuration.

In all the examples, the upper and lower bounds used for the eigenvalues $\lambda _i$ ($i=\{1,2,3\}$) (see (5.6)) are

$$\begin{aligned} \lambda _i^{lb}=0.01;\qquad \lambda _i^{ub}=12. \end{aligned}$$

With respect to the upper and lower bounds used for $\{\theta _1,\theta _2,\theta _3\}$ (see (5.6)), these are

$$\begin{aligned} \begin{aligned} \theta _1^{lb}&= 0;\qquad&\theta _2^{lb}&= 0;\qquad&\theta _3^{lb}&=0;\\ \theta _1^{ub}&= 2\pi ;\qquad&\theta _2^{ub}&= \pi ;\qquad&\theta _3^{ub}&=2\pi . \end{aligned} \end{aligned}$$

We have chosen these bounds since in the performed experiments, it is not expected that rotations of more than one loop take place, but of course the above bounds can be expanded if the geometry of the problem suggests so.

#### Beam-Like Applications

**Fig. 2:**
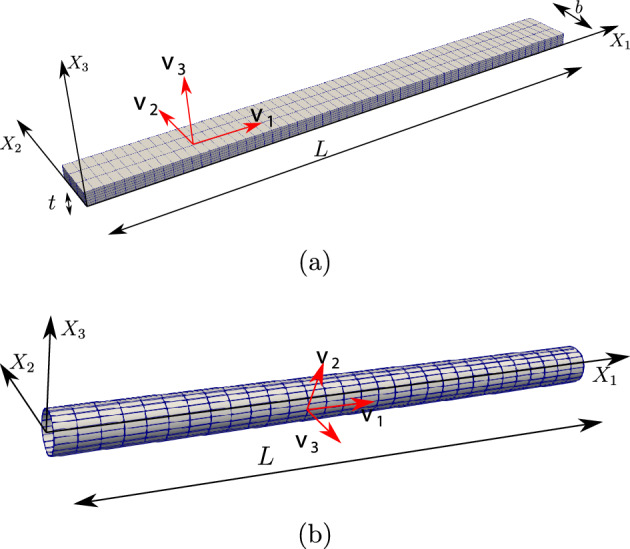
Geometry, finite element mesh and vectors $\{\varvec{v}_1,\varvec{v}_2,\varvec{v}_3\}$ parametrising the growth tensor $\varvec{C}_{\textrm{g}}$. **a** Rectangular section beam, with $\{L,b,t\}=\{1,1/10,1/50\}$. **b** Circular section beam with $\{L,R,t\}=\{30,1,1/20\}$. In both cases, *t* is the thickness of the beam

The first examples consider applications where the geometry of the undeformed domain $\Omega _0$ resembles that of a beam. In particular, we consider the rectangular section beam in Fig. 2a and the beam with circular cross-section in Fig. 2b. For both cases, the eigenvectors $\{\varvec{v}_1,\varvec{v}_2,\varvec{v}_3\}$ featuring in the definition of $\varvec{C}_{\textrm{g}}$ in (5.2) are defined as

$$\begin{aligned} \begin{aligned} \varvec{v}_1=\begin{bmatrix} 1{{,}}&0{{,}}&0 \end{bmatrix}^T\qquad \varvec{v}_2=\begin{bmatrix} 0 {{,}}&1 {{,}}&0 \end{bmatrix}^T\qquad \varvec{v}_3=\begin{bmatrix} 0 {{,}}&0 {{,}}&1 \end{bmatrix}^T \end{aligned} \end{aligned}$$

for the case in Fig. 2a and

$$\begin{aligned} \begin{aligned} \varvec{v}_1=\begin{bmatrix} 1 {{,}}&0 {{,}}&0 \end{bmatrix}^T\qquad \varvec{v}_2=\begin{bmatrix} 0 {{,}}&-\sin \theta {{,}}&\cos \theta \end{bmatrix}^T\qquad \varvec{v}_3=\begin{bmatrix} 0 {{,}}&\cos \theta {{,}}&\sin \theta \end{bmatrix}^T \end{aligned} \end{aligned}$$

for the case in Fig. 2b. In both cases, the boundary conditions are such that the displacements in $X_1=0$ are 0 in the three directions $\{\varvec{E}_1, \varvec{E}_2, \varvec{E}_3\}$ of the configuration $\{X_1, X_2, X_3\}$. Three target configurations, $\Omega _{\text {target}}=\varvec{\Phi }_d\left( \Omega _0\right)$, have been prescribed:

(i) *Shape morphing configuration 1*: rectangular cross-section beam with target configuration given by
  5.14 $$\begin{aligned} \varvec{\Phi }_d(\varvec{X}) = \begin{bmatrix} X_1 {{,}}&X_2 {{,}}&X_3 + 0.15L\sin \left( 2 \pi \frac{X_1}{L}\right) \end{bmatrix}^T . \end{aligned}$$
(ii) *Shape morphing configuration 2*: rectangular cross-section beam with target configuration given by
  5.15 $$\begin{aligned} \varvec{\Phi }_d(\varvec{X})=\begin{bmatrix} \frac{L}{2\pi }\sin \left( \frac{2\pi X_1}{L}\right) {{,}}&X_2 {{,}}&-\frac{L}{2\pi }\cos \left( \frac{2\pi X_1}{L}\right) \end{bmatrix}^T. \end{aligned}$$
(iii) *Shape morphing configuration 3*: circular cross-section beam with target configuration given by
  5.16 $$\begin{aligned} \varvec{\Phi }_d(\varvec{X})=\begin{bmatrix} -(R_f + \cos \theta )r \cos \left( \frac{2\pi X_1}{L_f} + \frac{\pi L}{4}\right) \\ (R_f + \cos \theta )r\sin \left( \frac{2\pi X_1}{L_f} + \frac{\pi L}{4}\right) -6\\ r\sin \theta + \frac{X_1L_f}{L}+2 \end{bmatrix}, \end{aligned}$$
  with $R_f=6$ and $L_f=4$, and with $(r,\theta )$ given by
  $$\begin{aligned} r=\sqrt{X_2^2+X_3^2},\qquad \tan \theta =\frac{X_3}{X_2}. \end{aligned}$$

**Fig. 3:**
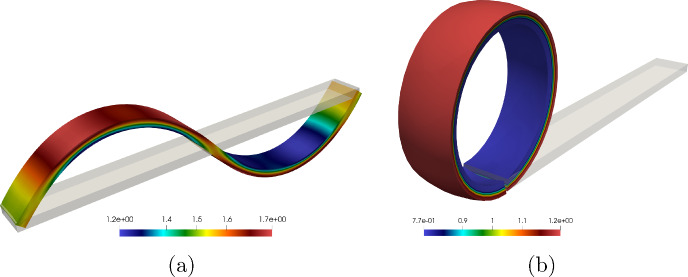
Representation of the computed optimal deformation $\varvec{\Phi }$ (indistinguishable from the target configuration) and the contour plot distribution of $\hat{\lambda }_1 \left( \varvec{\Phi }^{-1}(\varvec{x})\right)$, for the beam with rectangular section in Fig. 2a for target configurations: **a** equation (5.14); **b** equation (5.15). The translucid configuration represents the undeformed configuration $\Omega _0$

**Fig. 4:**
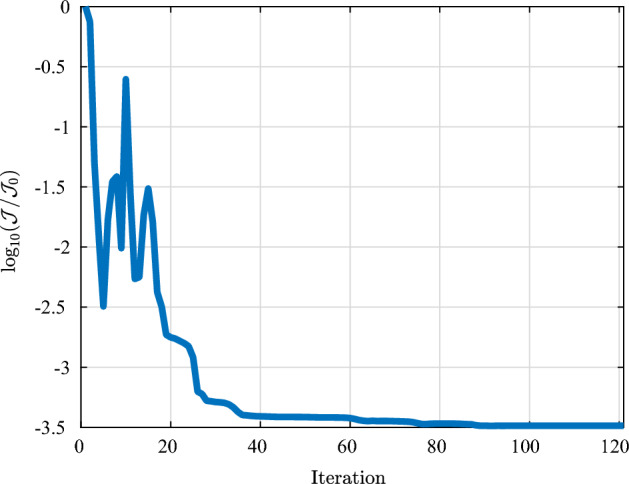
Evolution of the objective function with the number of iterations for the target configuration given in Eq. (5.14)

For the case of the rectangular cross-section beam in Fig. 2a, the final configurations attained at convergence are depicted in Fig. 3, corresponding with the optimal solutions that yield the closest growth-driven configurations to the target configurations denoted as *shape morphing configurations 1* and *2*. In addition, Fig. 4 depicts the evolution of the cost function for the case of the shape morphing configuration 1. The interior-point algorithm has been used as the optimization method.

**Fig. 5:**
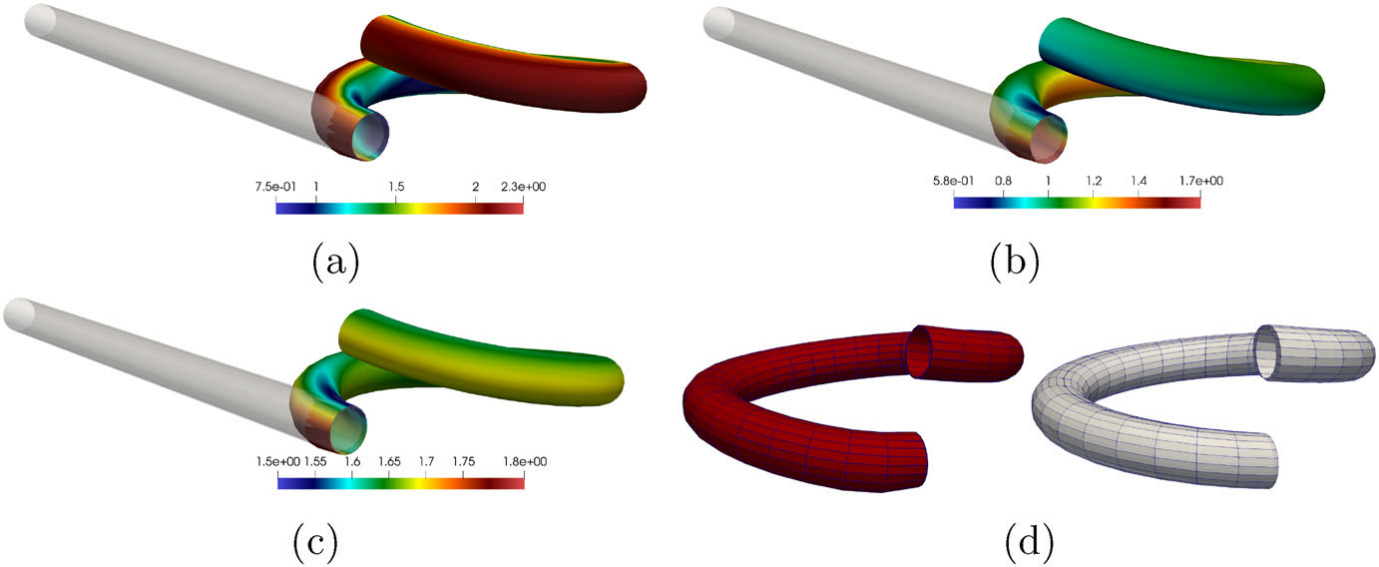
Representation of the computed optimal deformation $\varvec{\Phi }$ and contour plot distribution of **a** $\hat{\lambda }_1\left( \varvec{\Phi }^{-1}(\varvec{x})\right)$, **b** $\hat{\lambda }_2\left( \varvec{\Phi }^{-1}(\varvec{x})\right)$ and **c** $\hat{\lambda }_3\left( \varvec{\Phi }^{-1}(\varvec{x})\right)$ for the example with initial configuration depicted in Fig. 2. The translucid geometry represents the initial configuration. **d** Agreement between the target configuration (grey colour) and the deformed solid subjected to the optimal growth tensor (red) (Color figure online)

With regard to the circular cross-section beam in Fig. 2b, with target configuration given in Eq. (5.16), the final growth-driven configuration is displayed in Fig. 5, along with the contour plot distribution of the three design variables $\{\hat{\lambda }_1,\hat{\lambda }_2,\hat{\lambda }_3\}$. The tight agreement with respect to the target configuration initially prescribed in Eq. (5.16) is shown in Fig. 5d.

#### Shell-Type Applications

**Fig. 6:**
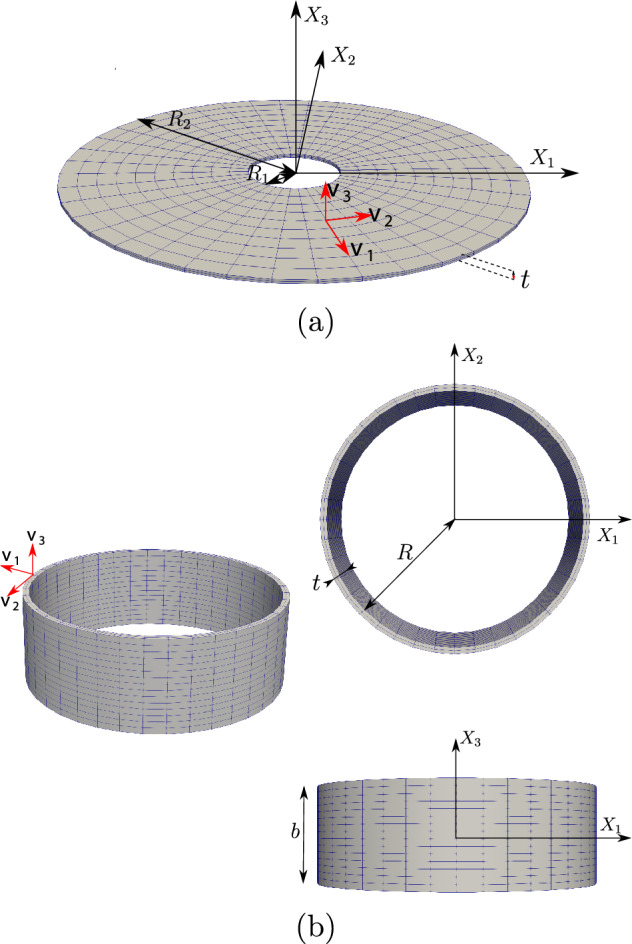
Geometry, finite element mesh and vectors $\{\varvec{v}_1,\varvec{v}_2,\varvec{v}_3\}$ parametrising the growth tensor $\varvec{C}_{\textrm{g}}$. **a** Disk with $\{R_1,R_2,t\}=\{0.2,1,0.8/50\}$. **b** Cylinder with $\{L,R,t\}=\{0.7,1,1/20\}$

Next, we consider the two undeformed configurations given in Fig. 6a and the beam with circular cross-section in Fig. 6b. For both cases, the eigenvectors $\{\varvec{v}_1,\varvec{v}_2,\varvec{v}_3\}$ are defined as

$$\begin{aligned} \begin{aligned} \varvec{v}_1=\begin{bmatrix} \cos \theta {{,}}&\sin \theta {{,}}&0 \end{bmatrix}^T\qquad \varvec{v}_2=\begin{bmatrix} -\sin \theta {{,}}&\cos \theta {{,}}&0 \end{bmatrix}^T\qquad \varvec{v}_3=\begin{bmatrix} 0 {{,}}&0 {{,}}&1 \end{bmatrix}^T . \end{aligned} \end{aligned}$$

In both cases, the boundary conditions are such that the displacements vanish in $X_3$ and $r=R_1$ (for Fig. 6a) and $r=R$ (for Fig. 6b) in the three directions $\{\varvec{E}_1, \varvec{E}_2, \varvec{E}_3\}$ of the configuration $\{X_1, X_2, X_3\}$. Two target configurations, $\Omega _{\text {target}} = \varvec{\Phi }_d\left( \Omega _0\right)$, have been prescribed:

(i) *Shape morphing configuration 4*: initial geometry given in Fig. 6a with target configuration given by
  $$\begin{aligned} \varvec{\Phi }_d(\varvec{X})=\begin{bmatrix} r\cos \theta {{,}}&r\sin \theta {{,}}&\frac{5}{8}(r-R_1) + X_3 \end{bmatrix}^T. \end{aligned}$$
(ii) *Shape morphing configuration 5*: initial geometry given in Fig. 6b with target configuration given by
  $$\begin{aligned} \varvec{\Phi }_d(\varvec{X})=\begin{bmatrix} (X_3+1)\cos \theta {{,}}&(X_3+1)\sin \theta {{,}}&\left( 6 - \frac{1}{8}(7 - 2(X_3+1))^2 - \frac{23}{8}\right) \end{bmatrix}^T, \end{aligned}$$
  with
  $$\begin{aligned} r=\sqrt{X_1^2+X_2^2},\qquad \tan \theta =\frac{X_2}{X_1} \end{aligned}$$
  in both configurations.

**Fig. 7:**
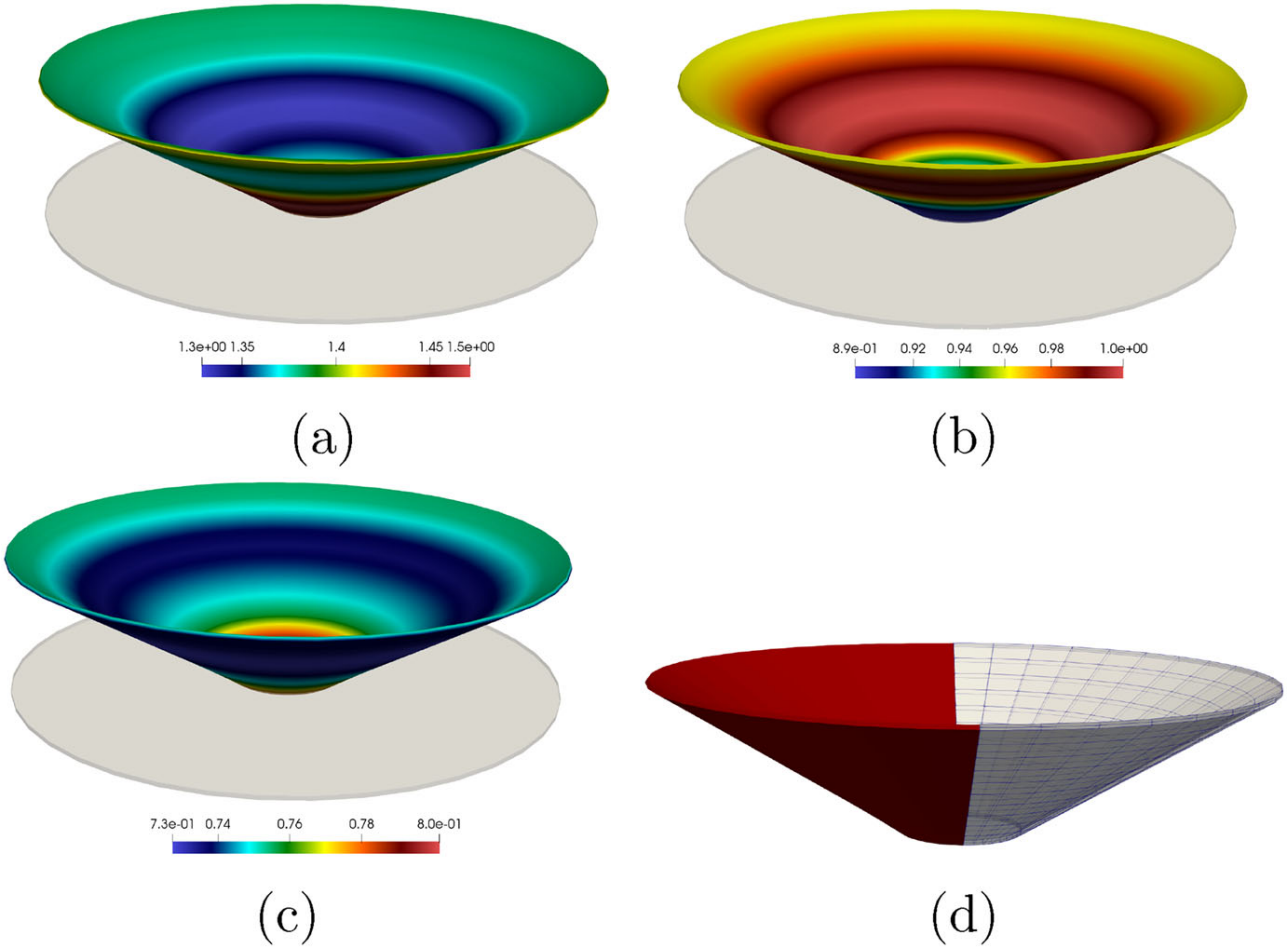
Representation of the computed optimal deformation $\varvec{\Phi }$ and contour plot distribution of **a** $\hat{\lambda }_1$, **b** $\hat{\lambda }_2$ and **c** $\hat{\lambda }_3$ for the example with initial configuration depicted in Fig. 6a. The translucid geometry represents the initial configuration. **d** Agreement between the target configuration (grey meshed domain) and the deformed solid subjected to the optimal growth tensor (red) (Color figure online)

For the case of the initial geometry in Fig. 6a, the final configuration attained can be observed in Fig. 7, corresponding with the optimal solutions that yield the closest growth driven configurations to the target configuration denoted as *shape morphing configuration 4*. It is worth empashising how the optimal solution is capable of, starting with a flat disk geometry, inducing a deformation of the continuum into the final conical shape illustrated in this figure. From Fig. 7d, the almost perfect match between the growth-driven and target configurations can be observed.

**Fig. 8:**
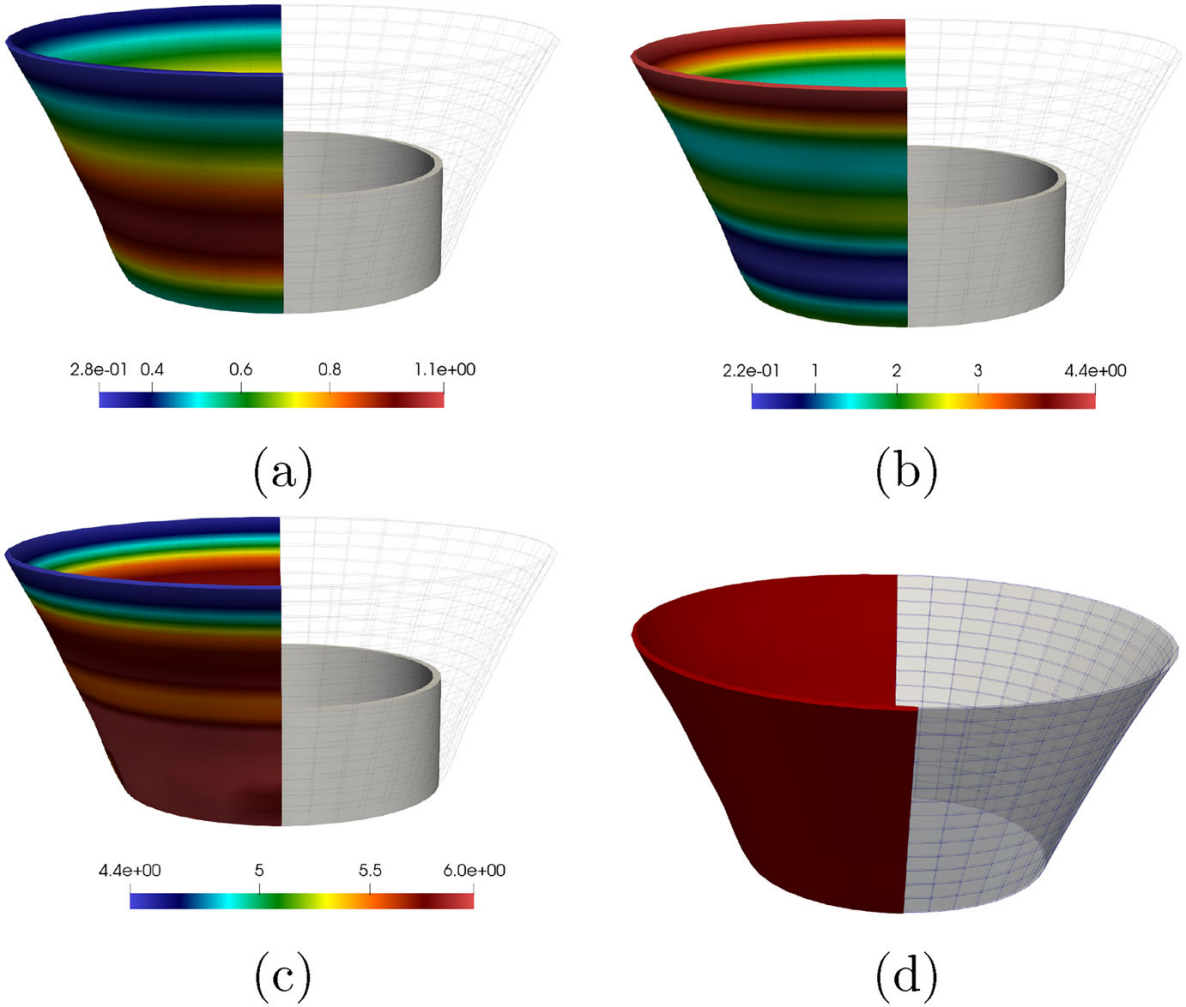
Representation of the computed optimal deformation $\varvec{\Phi }$ and contour plot distribution of **a** $\hat{\lambda }_1$, **b** $\hat{\lambda }_2$ and **c** $\hat{\lambda }_3$ for the example with initial configuration depicted in Fig. 6b. The grey domain represents the initial configuration. **d** Agreement between the target configuration (grey meshed domain) and the deformed solid subjected to the optimal growth tensor (red) (Color figure online)

Finally, for the case of the initial geometry in Fig. 6b, the final configuration attained can be observed in Fig. 8. In this case, attaining the target configuration entails a considerable enlargement of the initial geometry along the $X_3$ direction, in addition to a bending in the $X_1$ and $X_2$ directions, yielding the conical shape illustrated in this figure. Figure 8d shows the almost perfect match between the growth-driven and target configurations can be observed.

#### Cube to Cylinder Geometry

**Fig. 9:**
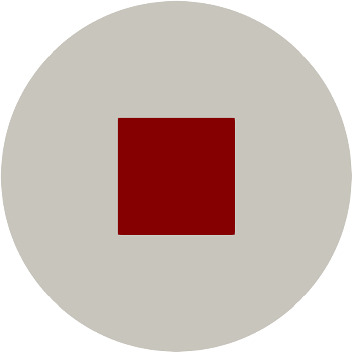
Undeformed configuration (red), representing a square of side 4. The circular geometry represents the target configuration, with diameter 12 (Color figure online)

The objective of this example is to evidence what was anticipated in the introductory part of Sect. 5.2. Specifically, although the examples shown in Sects. 5.2.1 and 5.2.2 have demonstrated that including only eigenvalues as design variables whilst maintaining the eigenvectors fixed throughout the optimisation process can yield extremely good results, this might not be the case for any predefined target configuration. In order to illustrate that, we consider now the initial and target configurations shown in Fig. 9. The problem requires a transformation from a cube into a cylinder, although the pictures are represented in 2D.

We solved the problem using two formulations:

- The first formulation involved exclusively the eigenvalues $\{\lambda _1,\lambda _2,\lambda _3\}$ as design variables, whilst holding the eigenvectors fixed throughout the optimisation to $\varvec{v}_1=\left[ 1,0,0\right] ^T$, $\varvec{v}_2=\left[ 0,1,0\right] ^T$ and $\varvec{v}_3=\left[ 0,0,1\right] ^T$.
- The second formulation considered both $\{\lambda _1,\lambda _2,\lambda _3\}$ and $\{\theta _1,\theta _2,\theta _3\}$ as design variables, the latter used to parametrise the eigenvectors $\{\varvec{v}_1,\varvec{v}_2,\varvec{v}_3\}$ according to (5.5).

**Fig. 10:**
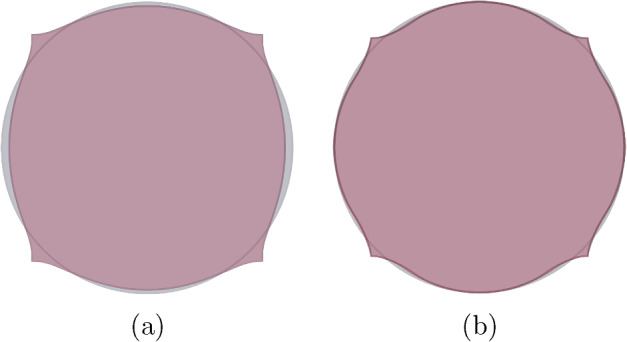
**a** Deformed configuration for the optimal solution yielded by formulation including only $\{\lambda _1,\lambda _2,\lambda _3\}$ as design variables whilst fixing $\varvec{v}_1=\left[ 1,0,0\right] ^T$, $\varvec{v}_2=\left[ 0,1,0\right] ^T$ and $\varvec{v}_3=\left[ 0,0,1\right] ^T$; **b** Deformed configuration for the optimal solution obtained by the formulation that includes both $\{\lambda _1,\lambda _2,\lambda _3\}$ and $\{\theta _1,\theta _2,\theta _3\}$ as design variables

Figure 10 includes the results yielded by both formulations. As expected, the deformed configuration resulting from the second formulation (including both $\{\lambda _1,\lambda _2,\lambda _3\}$ and $\{\theta _1,\theta _2,\theta _3\}$ as design variables) yields a significantly better approximation to the unattainable circular target configuration. This is corroborated by the values of the objective function $\rho _H$ attained by both formulations in the last optimisation iteration, when numerical convergence was observed. Specifically, the ratio between the values yielded by both formulations were

$$\begin{aligned} \frac{\rho _H \left( \Omega \left( \varvec{C}_{\textrm{g}}\left( \hat{\varvec{\lambda }} \right) \right) ,\Omega _{\text {target}}\right) }{\rho _H \left( \Omega \left( \varvec{C}_{\textrm{g}}\left( \hat{\varvec{\lambda }},\hat{\varvec{\theta }} \right) \right) ,\Omega _{\text {target}}\right) }=13.8889 , \end{aligned}$$

where the value in the numerator refers to the first formulation (only $\lambda _1,\lambda _2,\lambda _3$ as design variables).

**Fig. 11:**
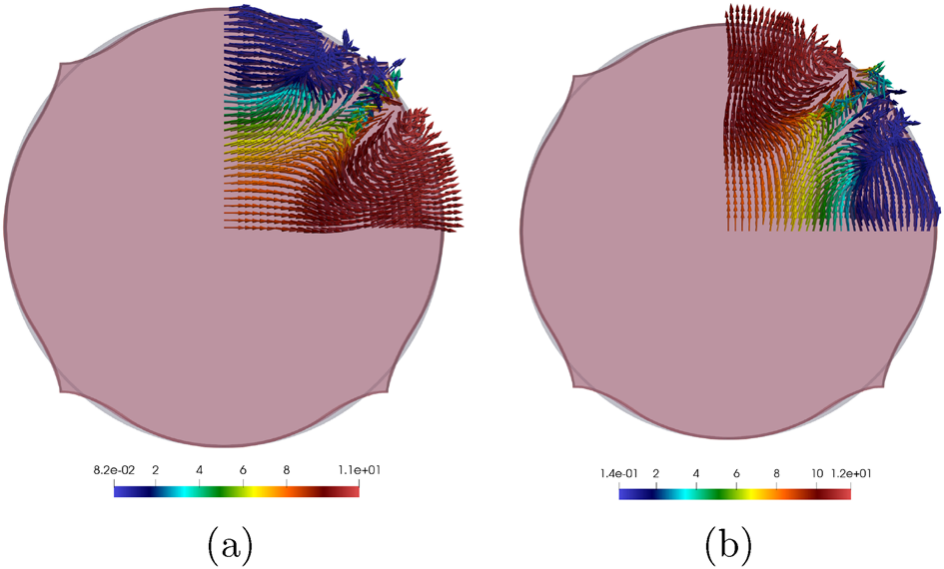
Deformed configuration in the optimal solution obtained by formulation including eigenvalues $\{\lambda _1,\lambda _2,\lambda _3\}$ and angular fields $\{\theta _1,\theta _2,\theta _3\}$ parametrising the eigenvectors. **a** Representation of the eigenvector $\varvec{v}_1$, where the colour of the vector is associated with the magnitude of $\lambda _1$; **b** Representation of the eigenvector $\varvec{v}_2$, where the colour of the vector is associated with the magnitude of $\lambda _2$

Finally, Fig. 11 illustrates the optimal solution obtained for the eigenvectors $\varvec{v}_1(\theta _1,\theta _2,\theta _3)$ and $\varvec{v}_2(\theta _1,\theta _2,\theta _3)$. This figure demonstrates the necessity to modify spatially these eigenvectors in order to yield the observed higher flexibility.
